# Assessment of soil heavy metals for eco-environment and human health in a rapidly urbanization area of the upper Yangtze Basin

**DOI:** 10.1038/s41598-018-21569-6

**Published:** 2018-02-19

**Authors:** Zhongmin Jia, Siyue Li, Li Wang

**Affiliations:** 1 Key Laboratory of Eco-environments of the Three Gorges Reservoir Region, Ministry of Education, College of Life Science, Southwest University, Chongqing, 400715 China; 2 Southeast Sichuan Geological Group, Chongqing Bureau of Geology and Minerals Exploration, Chongqing, 400038 China; 3 Chongqing Institute of Green and Intelligent Technology, Chinese Academy of Sciences, Chongqing, 400714 China

## Abstract

Soil pollution with heavy metals (HMs) has been attracting more and more interests, however, assessment of eco-environmental and human risks particularly in a rapidly urbanization area (the upper Yangtze) remains limited. Multiple modern indices were firstly performed for complete risk assessment of eco-environment and human health based on a high-spatial-resolution sampling. Averages of HMs were far below grade II threshold level of the Chinese Environmental Quality standards for soils, whereas Cd, As and Hg considerably exceeded the local background values. EF suggested overall moderate enrichments of Cd and Se, resulting in soils uncontaminated to moderately contaminated with them. Potential ecological risk index showed significant differences among Counties that were characterized by moderate risk. However, several sites were moderately to heavily contaminated with As, Cd and Hg by Igeo, resulting in that these sites were categorized as “considerable risk”, or “high risk”. Moreover, children were more susceptible to the potential health risk irrespective of the carcinogenic or non – carcinogenic risk. There were no significant carcinogenic and non – carcinogenic risks for adults, children however showed significant non – carcinogenic effect. Our first assessment provided important information for policy making to reduce the potential effects of soil contamination on human and eco-environment.

## Introduction

Due to the natures of ubiquity, toxicity at a trace level, bioaccumulation and persistence, elevated heavy metals (HMs) in soil environment and thus soil contamination with HMs has been attracting much attention worldwide^1–5^. Moreover, HMs that have been substantially accumulated in soils can release to other ecosystems, such as groundwater, rivers, atmosphere and crops, and consequently are hazardous to human beings and ecosystems^2,6,7^. HMs, the naturally ubiquitous substances in soils, could be both natural (lithogenic inputs *via* weathering of parent materials and bedrocks) and anthropogenic in origin^8,9^. Nevertheless, obviously worldwide enrichments of HMs in soils are primarily due to a variety of human activities^4^. In urban areas, anthropogenic sources of soil HMs include traffic emissions (vehicle exhaust, tire wear, brake lining wear, etc), industrial discharges (power plant, chemical plant, coal combustion, metallurgical industry, etc), and municipal wastes^10^. The main sources of HMs in agricultural soils are derived from mining, smelting, vehicle exhaust, as well as applications of pesticides and fertilizers^11,12^.

Soil heavy metal (HM) pollution has become a severe issue in many parts of the world^3,8,13^, and also has been both serious and widespread in China following the rapid socio-economic development^4,14^. Numerous studies associated with HM contamination in soils therefore have focused on levels and eco-environmental risk assessments of HMs^2,15,16^. Multiple indices such as geoaccumulation index (Igeo)^2,11^ and enrichment factor (EF)^17^ were widely used for environmental risk assessment, while Hankson potential ecological risk index (RI) for eco-risk assessment^10,15,18^. The former is based on the ratio of measured element to a reference value, whist the later takes into consideration the toxic response factor of element. Generally, two typical index methods are used for reliable evaluation of eco-environment risks by soil HMs.

Efforts have been made on health risk assessment of soil HMs, however, compared with studies involving investigation and environmental risk of soil HMs, studies that have been conducted for human health risk assessment in urban soils need to be strengthened^2,16^. Previous research reported excess intake of HMs from soils can result in numerous diseases^19^. For example, chronic exposure to As can lead to dermal lesions, skin cancer, peripheral neuropathy, and peripheral vascular disease^20^, while chronic ingestion of Cd can have adverse effects such as prostatic proliferative lesions, bone fractures, kidney dysfunction, hypertension lung cancer, and pulmonary adenocarcinomas^21^. Excessive intake of Pb can damage the skeletal, circulatory, nervous, enzymatic, endocrine, and immune systems^22^. Thus, human health risk *via* direct exposure to soil HMs should not be ignored. Due to diverse landscape and heterogeneous human activities, soil pollution levels with HMs and their effects on eco-environment and humans are understandably quite variable in different area. It is necessary to carry out risk evaluations of eco-environment and human exposure to soil HMs everywhere to explore the adverse effects posed by HMs in soils and to protect human health.

**Figure 1:**
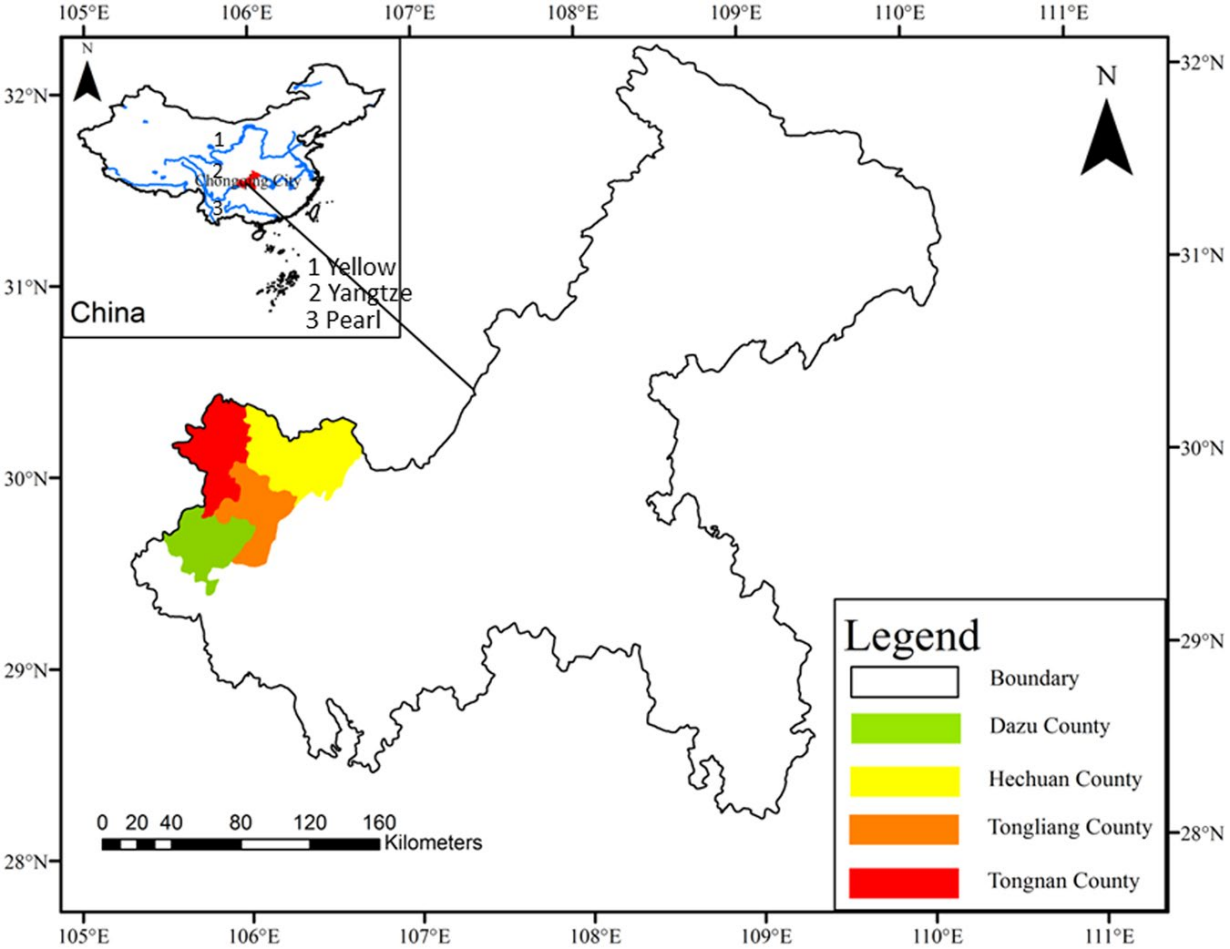
Location of sampling site in four Counties of the upper Yangtze (The data set is provided by Data Center for Resources and Environmental Sciences, Chinese Academy of Sciences (RESDC), http://www.resdc.cn; The data ArcMap 10.3 software is used to create Fig. 1 with Figure legends, and the software can be accessible *via* the link https://blogs.esri.com/esri/arcgis/2014/12/10/arcgis-10-3-the-next-generation-of-gis-is-here/).

Increasing studies have been conducted on toxic metal concentrations, contamination assessment^4,23^, health risk assessment and source identification of HMs particularly in urban and agricultural soils in China^9,24^. However, a very limited number of risk assessment studies have been undertaken regarding pollution levels and health risks of HMs in the soils of the upper Yangtze Basin^2,4^. This represents a knowledge gap for understanding the potential effects of soil HMs on human health and eco-environment. Chongqing, one of the municipalities in China, is experiencing rapid urbanization and industrialization. The west part of Chongqing is also an important agricultural base in Southwest (SW) China in the upper Yangtze. To better understand the potential risks of soil HMs in a rapid urbanization area, a pilot study was performed in several typical counties of the upper Yangtze (Fig. 1). The study aimed at (1) exploring eco-environmental risks of HMs in soils using multiple indices, and (2) identifying priority pollutants and regions of concern using non-carcinogenic and carcinogenic health risk assessment models associates with local residents exposed to HMs in soils. We test the hypothesis that similar to other urban soils, HMs pose harmful effects on eco-environment and human health, and these harmful effects are lower because of low urbanization. This study will be helpful for pollution control in relation to human health risk.

## Results and Discussion

### Concentrations of HMs

Basic statistics of eight priority HMs (As, Cd, Cr, Cu, Hg, Ni, Pb and Zn) and other elements are shown in Tables 1 and 2. There were significant changes in individual element among counties and each county showed similar trends of elements (p < 0.05 by ANOVA). As expected, Si was the most abundant element, followed by Al and Fe, which was consistent with their contents in the earth crust. Hg had the lowest concentration, Cd and Se showed the second lowest levels. In general, averages and 95% CI of HM concentrations were all below the Grade II criterion of the Chinese Environmental Quality standards for soils^25^. Among the sampling sites, maximal concentrations of As and Cu slightly surpassed their corresponding limits, the highest concentrations of Cd, Hg and Ni were about 2.6, 3.6, and 1.9 times their corresponding standard limits (Table 2). Concentrations of As in 4 samples (0.24%) (2 samples in HC, 1 in TL and 1 in DZ), Cu in 3 samples (0.18%) (3 samples in HC), Cd in 27 samples (1.63%) (3 samples in TC, 2 in TN, 3 in TL, 19 in DZ), Hg in 9 samples (0.54%) (5 in HC, 1 in TN, 2 in TL, 1 in DZ), Ni in 68 samples (4.09%) (29 in HC, 29 in TN, 8 in TL, 2 in DZ) were beyond their target values of China’s guidelines for soils. The results compared with the Chinese soil standard demonstrated that no obvious HM pollution was found in this area.

**Table 1:** Soil metal concentrations in four typical counties of Chongqing in the upper Yangtze Basin, China.

		n	Mean	S.D	S.E	95% CI		Min.	Max.
						LB	UB		
As	HC	582	5.48	3.42	0.14	5.20	5.76	1.20	31.00
	TN	385	7.29	2.53	0.13	7.04	7.54	1.75	14.61
	TL	337	5.75	3.41	0.19	5.39	6.12	1.57	27.40
	DZ	360	6.66	2.93	0.15	6.36	6.97	1.77	32.77
Cd	HC	582	0.301	0.077	0.003	0.294	0.307	0.130	1.390
	TN	385	0.345	0.058	0.003	0.339	0.351	0.200	0.900
	TL	337	0.323	0.078	0.004	0.314	0.331	0.110	0.720
	DZ	360	0.375	0.145	0.008	0.360	0.390	0.130	1.570
Cr	HC	582	71.63	12.21	0.51	70.64	72.63	44.50	144.40
	TN	385	83.44	9.28	0.47	82.51	84.37	59.90	103.40
	TL	337	72.00	12.50	0.68	70.66	73.34	40.00	115.30
	DZ	360	76.48	9.30	0.49	75.52	77.45	43.10	99.60
Cu	HC	582	25.96	11.83	0.49	25.00	26.92	9.10	106.50
	TN	385	30.09	4.91	0.25	29.59	30.58	13.60	54.00
	TL	337	25.71	7.54	0.41	24.91	26.52	6.80	83.20
	DZ	360	26.55	4.74	0.25	26.06	27.04	9.20	40.50
Hg	HC	581	0.082	0.092	0.004	0.074	0.089	0.010	1.600
	TN	385	0.054	0.042	0.002	0.050	0.058	0.020	0.550
	TL	337	0.096	0.128	0.007	0.082	0.110	0.020	1.790
	DZ	360	0.075	0.057	0.003	0.069	0.081	0.010	0.540
Ni	HC	582	32.12	9.89	0.41	31.32	32.93	11.60	96.39
	TN	385	41.58	6.88	0.35	40.89	42.27	26.00	57.40
	TL	337	32.80	8.97	0.49	31.84	33.76	12.10	78.20
	DZ	360	35.79	6.44	0.34	35.12	36.46	15.20	50.80
Pb	HC	580	26.93	2.94	0.12	26.69	27.17	19.80	55.00
	TN	385	27.42	2.26	0.12	27.19	27.65	19.60	44.40
	TL	337	28.49	3.19	0.17	28.15	28.83	18.10	45.70
	DZ	360	29.41	2.91	0.15	29.10	29.71	21.90	59.30
Zn	HC	582	82.79	15.94	0.66	81.50	84.09	35.00	238.50
	TN	385	95.28	11.55	0.59	94.12	96.44	61.10	137.50
	TL	337	84.83	18.06	0.98	82.89	86.76	36.50	226.40
	DZ	360	91.20	13.11	0.69	89.84	92.56	39.70	152.10
Co	HC	582	14.89	4.67	0.19	14.51	15.27	4.70	47.93
	TN	385	15.73	1.84	0.09	15.54	15.91	10.16	19.53
	TL	337	14.03	3.34	0.18	13.67	14.39	4.60	36.10
	DZ	360	15.19	2.05	0.11	14.97	15.40	6.41	20.85
Mn	HC	582	561.91	153.31	6.35	549.43	574.39	102.30	1433.00
	TN	385	624.32	96.13	4.90	614.68	633.95	313.10	1158.00
	TL	337	533.93	148.19	8.07	518.06	549.81	119.80	1144.00
	DZ	360	554.34	126.62	6.67	541.22	567.47	155.10	821.70
Mo	HC	582	0.592	0.275	0.011	0.570	0.615	0.300	2.440
	TN	385	0.944	0.262	0.013	0.918	0.971	0.390	2.460
	TL	337	0.639	0.248	0.014	0.612	0.665	0.320	1.960
	DZ	360	0.772	0.263	0.014	0.745	0.799	0.330	2.810
Sr	HC	582	153.88	42.02	1.74	150.46	157.30	39.00	294.50
	TN	385	131.84	26.95	1.37	129.14	134.54	76.50	220.80
	TL	337	137.53	51.01	2.78	132.07	143.00	42.90	392.40
	DZ	359	131.71	52.16	2.75	126.29	137.12	42.80	638.80
Ti	HC	582	4559.8	1493.9	61.9	4438.1	4681.4	3570.0	16275.4
	TN	385	4304.9	218.2	11.1	4283.0	4326.7	3603.5	5266.3
	TL	337	4314.1	688.1	37.5	4240.3	4387.8	3196.8	9956.0
	DZ	360	4359.6	309.0	16.3	4327.6	4391.6	3501.1	5890.3
Sb	HC	582	0.589	0.336	0.014	0.561	0.616	0.230	7.020
	TN	385	0.654	0.140	0.007	0.640	0.668	0.120	1.370
	TL	337	0.624	0.407	0.022	0.580	0.667	0.190	7.200
	DZ	360	0.645	0.171	0.009	0.627	0.662	0.360	2.540
Sc	HC	582	10.72	2.49	0.10	10.52	10.93	6.00	29.85
	TN	385	11.77	1.94	0.10	11.58	11.97	6.87	15.54
	TL	337	10.62	2.00	0.11	10.40	10.83	5.70	21.00
	DZ	360	11.09	1.55	0.08	10.93	11.25	6.71	14.50
Se	HC	582	0.230	0.120	0.005	0.221	0.240	0.100	1.320
	TN	385	0.180	0.068	0.003	0.173	0.187	0.100	1.020
	TL	337	0.237	0.093	0.005	0.227	0.247	0.090	0.720
	DZ	360	0.216	0.064	0.003	0.209	0.222	0.120	0.580
Sn	HC	582	2.87	0.53	0.02	2.82	2.91	2.10	9.60
	TN	385	2.82	0.45	0.02	2.78	2.87	1.80	6.80
	TL	337	2.83	0.38	0.02	2.79	2.87	1.80	3.80
	DZ	360	2.97	0.41	0.02	2.93	3.01	2.10	4.50

S.D.- standard deviation.

S.E.- Standard error.

95% CI-95% Confidence Interval for Mean.

LB Lower Bound.

UB Upper Bound.

Min. Minimum.

Max. Maximum.

**Table 2:** Total averages of soil metals and comparisons with background values, element contents in the upper continent crust, and well as China’s standards (unit in mg/kg).

	Mean	S.D	S.E	95% CI		Min.	Max.	Background^a^	UCC^b^	CEQS^c^
				LB	UB					
As	6.21	3.21	0.08	6.06	6.36	1.20	32.77	5.00	2.00	25
Cd	0.331	0.097	0.002	0.327	0.336	0.110	1.570	0.110	0.102	0.60
Cr	75.49	12.03	0.29	74.91	76.07	40.00	144.40	80.00	35.00	200
Cu	26.99	8.59	0.21	26.58	27.41	6.80	106.50	26.00	14.30	100
Hg	0.077	0.087	0.002	0.073	0.081	0.010	1.790	0.060	0.056	0.50
Ni	35.24	9.18	0.23	34.80	35.68	11.60	96.39	32.00	18.60	50
Pb	27.90	3.00	0.07	27.75	28.04	18.10	59.30	26.00	17.00	300
Zn	87.91	15.77	0.39	87.16	88.67	35.00	238.50	80.00	52.00	250
Co	14.98	3.45	0.08	14.81	15.14	4.60	47.93	15.00	11.60	40
Mn	569.1	138.8	3.4	562.4	575.7	102.3	1433.0	615.0	527.0	
Mo	0.722	0.298	0.007	0.708	0.736	0.300	2.810	0.500	1.40	
Sr	140.7	44.7	1.1	138.5	142.8	39.0	638.8	128.0	316.0	
Ti	4407.7	959.0	23.5	4361.6	4453.8	3196.8	16275.4	4893.0	3117.0	
Sb	0.623	0.290	0.007	0.609	0.637	0.120	7.200	0.700	0.3	10
Sc	11.02	2.13	0.05	10.92	11.13	5.70	29.85	12.00	7.0	
Se	0.217	0.096	0.002	0.212	0.222	0.090	1.320	0.100	0.083	3
Sn	2.87	0.46	0.01	2.85	2.89	1.80	9.60	3.10	2.50	
Si	296.04	21.32	0.52	295.02	297.07	244.40	366.89	302.30	303.48	
Al	77.49	4.08	0.10	77.30	77.69	55.64	98.79	78.10	77.44	
Fe	37.30	6.38	0.16	36.99	37.60	14.21	85.33	38.90	30.89	

a-Chongqing background values.

b-upper continent crust.

c-Chinese environmental quality standard for soils (grade II: 6.5 < pH < 7.5; GB15618–1995) (CEPA 1995).

However, in comparison with the soil background values in Chongqing (Table 2), the concentrations of Cr, Cu, Ni, Pb and Zn were of the same order of magnitude, As and Hg were slightly higher, which were 1.24 and 1.28 times higher than their respective background values, Cd, however, was 3.01 times greater than its background value. When compared with global background values (Table 2), the concentrations of the eight priority HMs were obviously higher, which was especially true for As, Cd, Cr, which were 3.1, 3.3, and 2.2 times higher than their respective background levels. The excessive loadings of HMs respective to background values might be a consequence of anthropogenic activities.

Compared with concentrations of eight priority HMs in urban soils of different cities in Chin that was collated by Luo *et al*.^4^, concentrations of HMs with an exception Cr in our study were much lower than averages of 21 cities in China^4^. For example, Hg concentration was 22% the average of 21 cities, this ratio was around 27% for Cu, and 35% for Ni^4^. Moreover, concentrations of HMs were especially high in Changsha (As (32.8 mg/kg) and Cd (6.90 mg/kg)), Jinchang (Cr (197 mg/kg), Cu (1226 mg/kg) and Ni (910 mg/kg)), and Baoji (Pb (25380 mg/kg) and Zn (1964 mg/kg))^2,4^. These specific areas are old industrial cities with extensive mining of metals and smelting operations. The comparison above highlighted the variability in HM concentrations among different regions, as well as the anthropogenic effects on heavy metal enrichment in soils Figure 1.

### Environmental risk assessment

**Figure 2:**
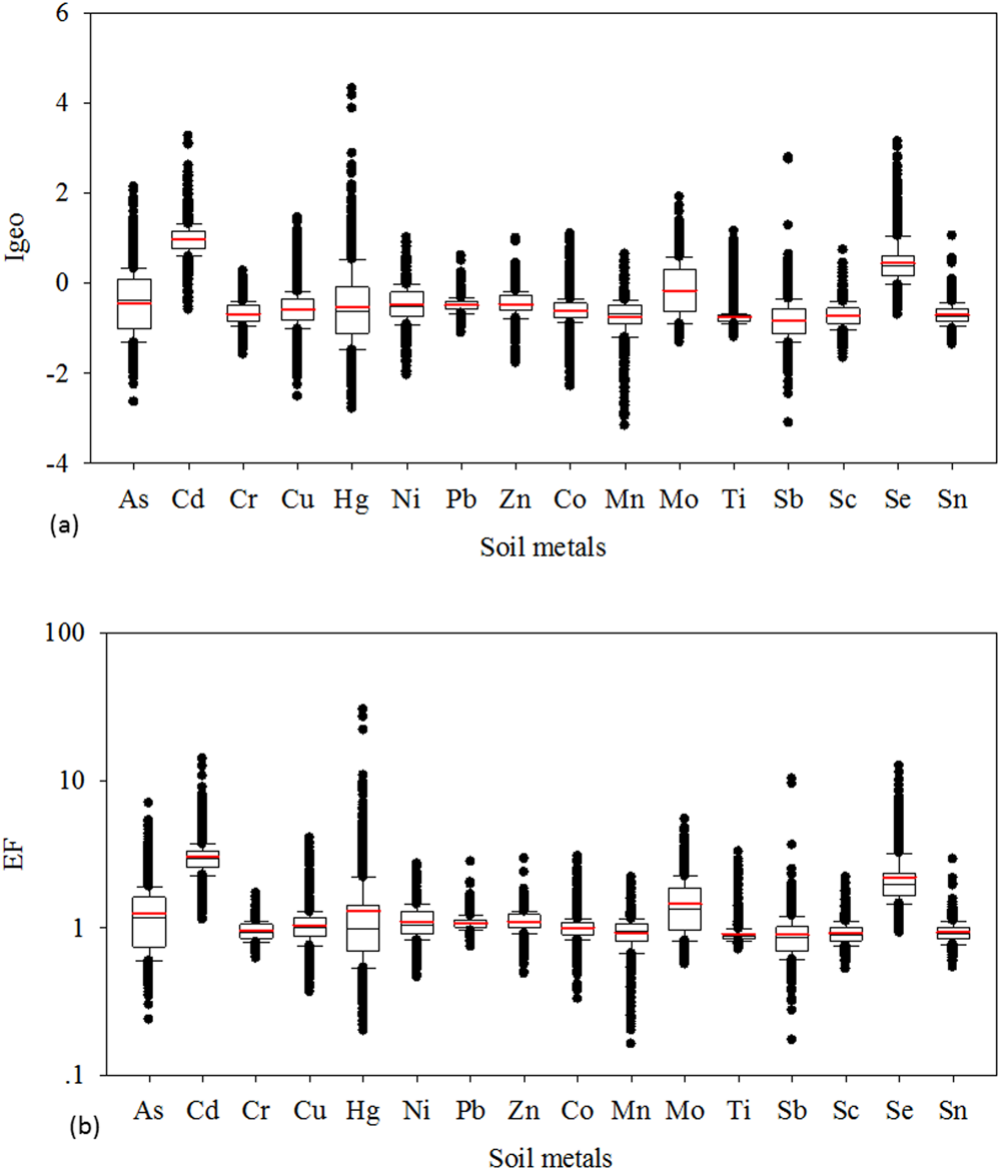
Pollution level of eight priority HMs and other trace elemen**ts** in the study area (Boxplots of Igeo (**a**) and EF (**b**)) (the black horizontal line represents the median, and the red horizontal line presents the mean. The box represents the 25^th^–75^th^ percentiles, and the whiskers represent the 10^th^–90^th^ percentiles). (For interpretation of the references to color in this figure legend, the reader is referred to the web version of this article).

Statistics of I_geo_ and EF of HMs are deciphered in Fig. 2, and Tables S1, S2 and S3. In general, Cr, Mn, Ti, Sb, Sc and Sn appeared to be the least contaminated elements, while Cd and Se had the highest I_geo_ values (Fig. 2a). Ranges in I_geo_ values for individual element were very wide, demonstrating the variability of soil properties and pollution sources of HMs. Among the counties, Tongnan showed higher I_geo_ values for As, Cr, Cu, Ni, Zn, Co, Mn, Mo, Sb and Sc (Table S1). Moreover, the mean values of I_geo_ values for Cd and Se were positive, while others had negative values of I_geo_. Thus, soils in this area were uncontaminated to moderately contaminated by Cd and Se, and soils were uncontaminated by other HMs. However, we needed to highlight that counties Tongnan and Dazu showed mean I_geo_ values of Cd greater than 1, suggesting that Cd fell into the category of “moderately contaminated”. We also found some sites with I_geo_ values greater than 2, thus, soils in several sites were moderately to heavily contaminated by As, Cd and Hg (Table S2). Particularly, Hg in several sites fell into the category of “heavily to extremely contaminated” in Hechuan and Tongliang (Table S2).

In comparison to I_geo_ of HMs in urban soils in other cities of China collated by Wei and Yang^2^, our values were much smaller. This was specially true for Pb and Cd. Wei and Yang^2^ reported that Cd fell into the category of “moderately to heavily contaminated” in urban soils, and soils were heavily contaminated by Pb in China.

EF showed consistent results with geo-accumulation index. Cd and Se showed highest averages of EF (>2) (Fig. 2b), demonstrating moderate enrichments of Cd and Se, others, however, showed minimal enrichments, as reflected by their EF levels below 2 (Fig. 2b). Similar to results by I_geo_, there were several sites with EF values of As, Cd, and Hg greater than 5, indicating their significant enrichments. Therefore, overall moderate enrichments of Cd and Se, as well as significant enrichments of As, Cd and Hg in some sites may be an indication of the influence of anthropogenic activities. Luo *et al*.^4^ reported moderate enrichments of Cd and Pb, and significant enrichment of Hg for urban soils in China, confirming broad enrichments of several HMs in urban soils by anthropogenic inputs, and highlighting serious pollutions of soil HMs in China^2,4^.

### Potential ecological risk assessment

**Figure 3:**
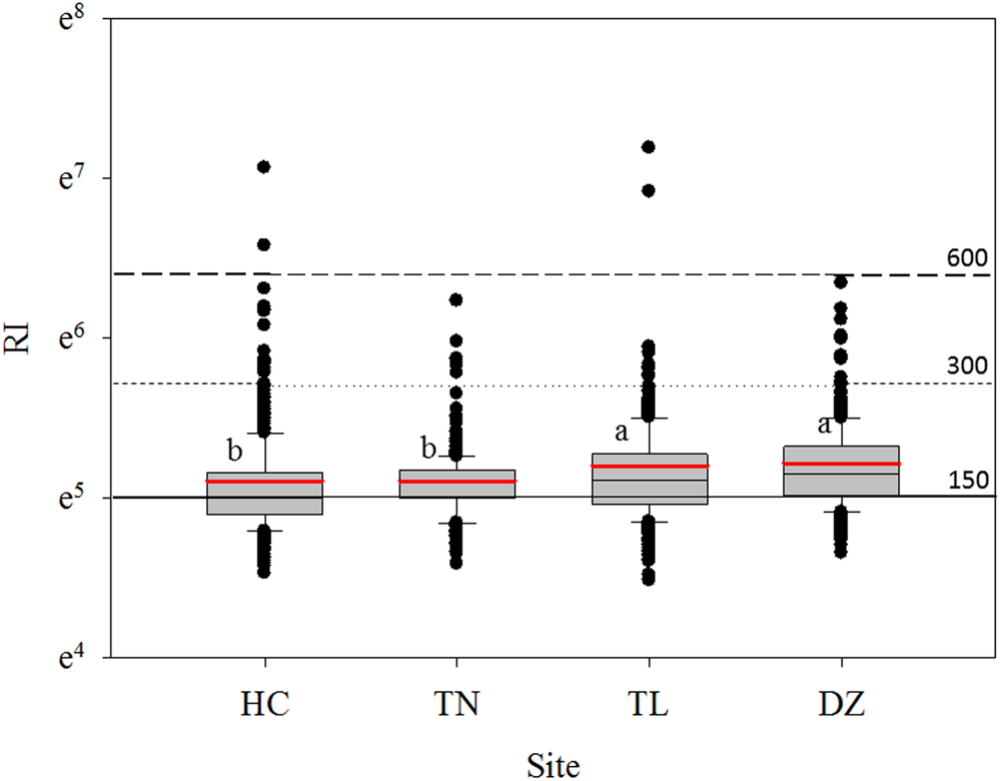
Boxplot of RI in the four counties of the upper Yangtze Basin, China (different letters show significant differences at p < 0.05 by ANOVA) (symbols for boxplot are similar to Fig. 2).

RI levels showed significant differences among counties, and its averages followed the descending order as DZ (184.9 ± 57.6; Mean ± S.D.) ≈ TL (182.0 ± 90.8) > TN (165.6 ± 36.9) ≈ HC (165.4 ± 71.3) (Fig. 3), demonstrating moderate risk of HMs. 51.0% of the sampling sites in the HC were classified as causing a low potential ecological risk, while 24.2–32.0% of sampling sites were low risk in other three counties. TN and DZ had 72% of sites that were categorized as “moderate risk”, 65% were moderate risk in TL, while 46% were moderate risk in HC. It should be noticeable that 2.7%, 1.3%, 2.4% and 3.6% of sites in the HC, TN, TL and DZ were categorized as “considerable risk”, as well as 0.3% and 0.6% of sites in HC and TL were classified as “high risk”, respectively.

### Human health risk assessment

**Figure 4:**
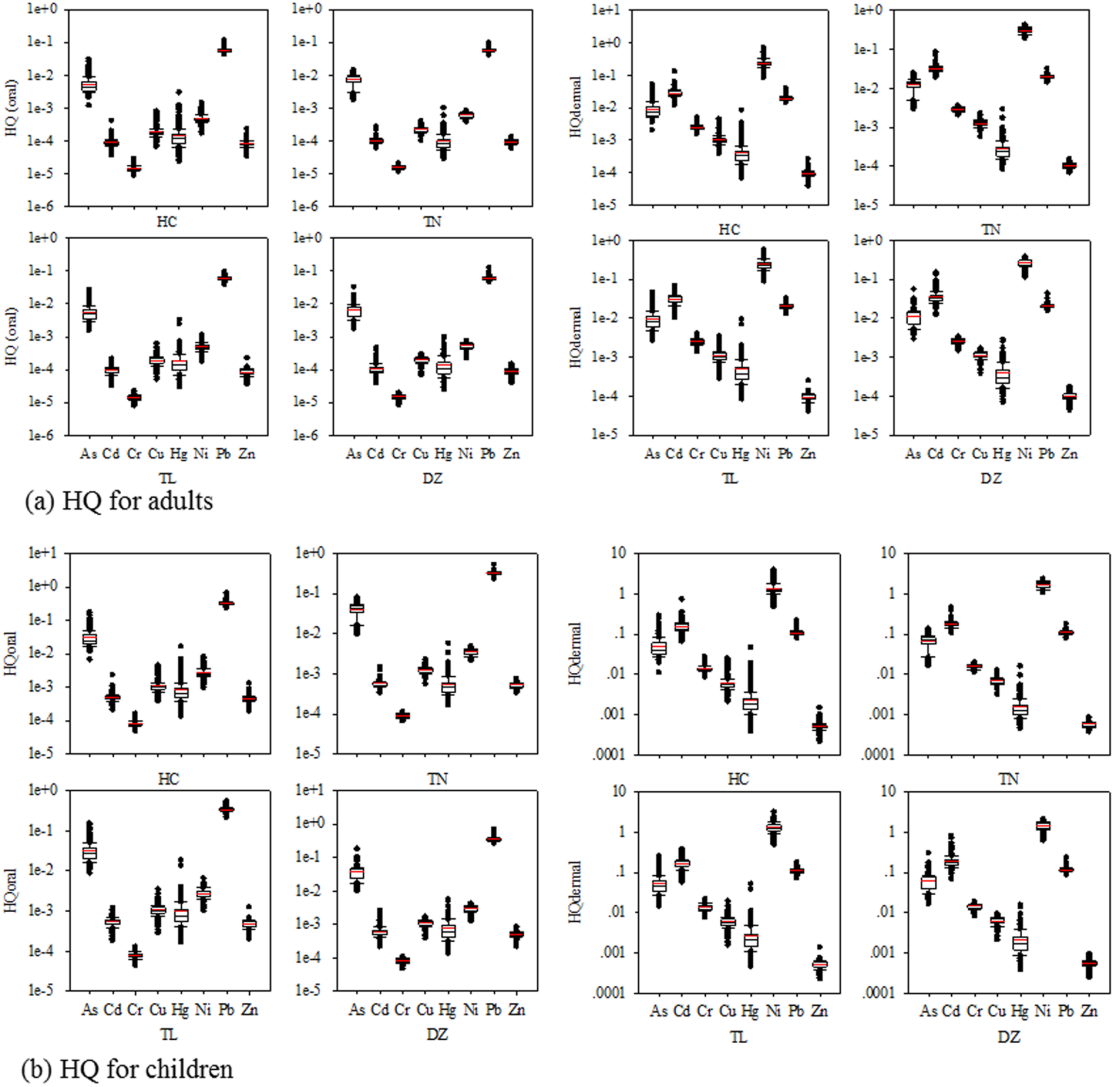
HQ_oral_ (the sum of ingestion and inhalation) and HQ_dermal_ for adults (**a**) children (**b**) exposure to HMS in soils of each County (symbols for boxplot are similar to Fig. 2).

**Figure 5:**
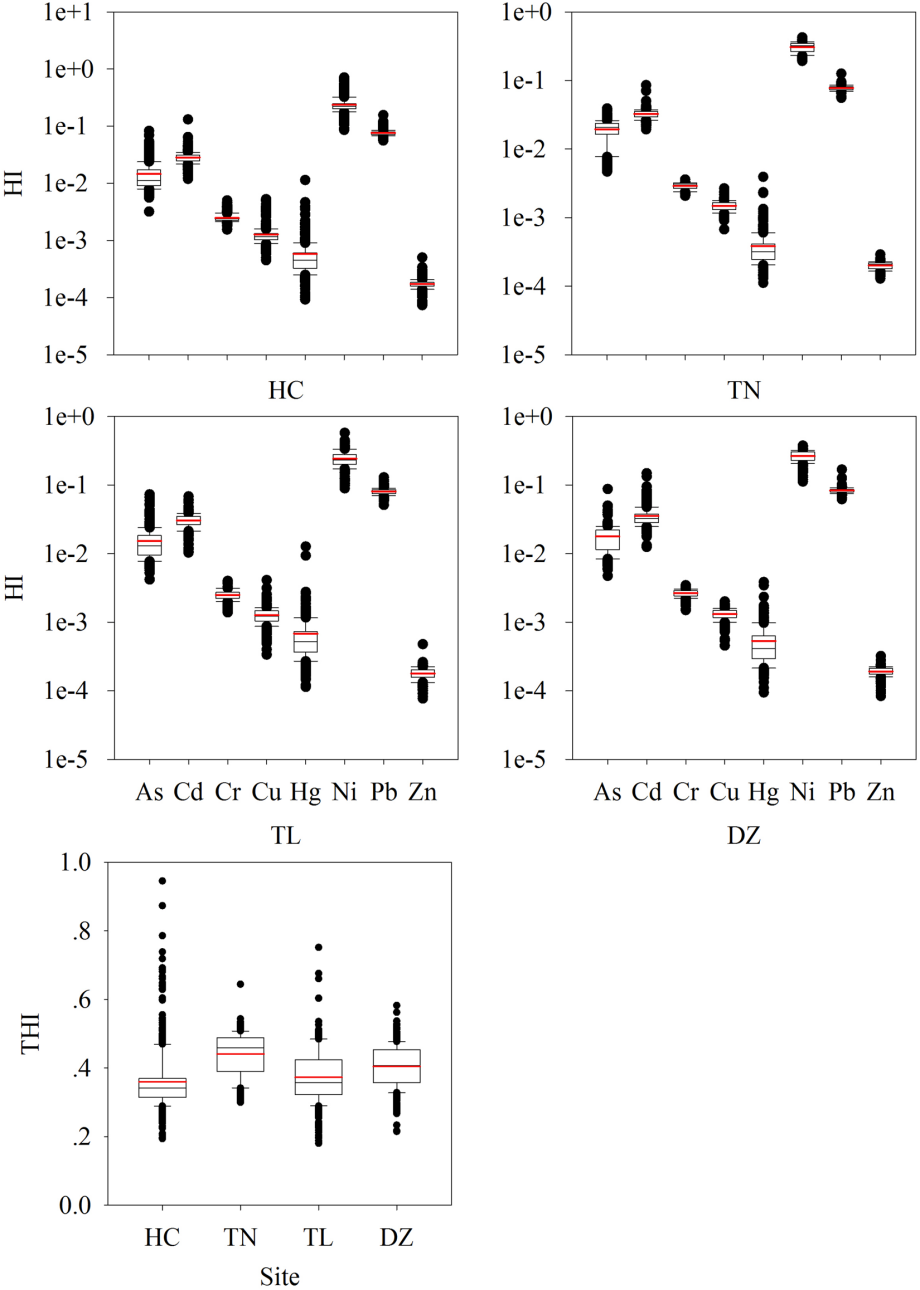
HI for adults exposure to HMs in soils of each County (THI for each County showed significant differences, p < 0.001 by Mann-Whitney U text) (symbols for boxplot are similar to Fig. 2).

**Figure 6:**
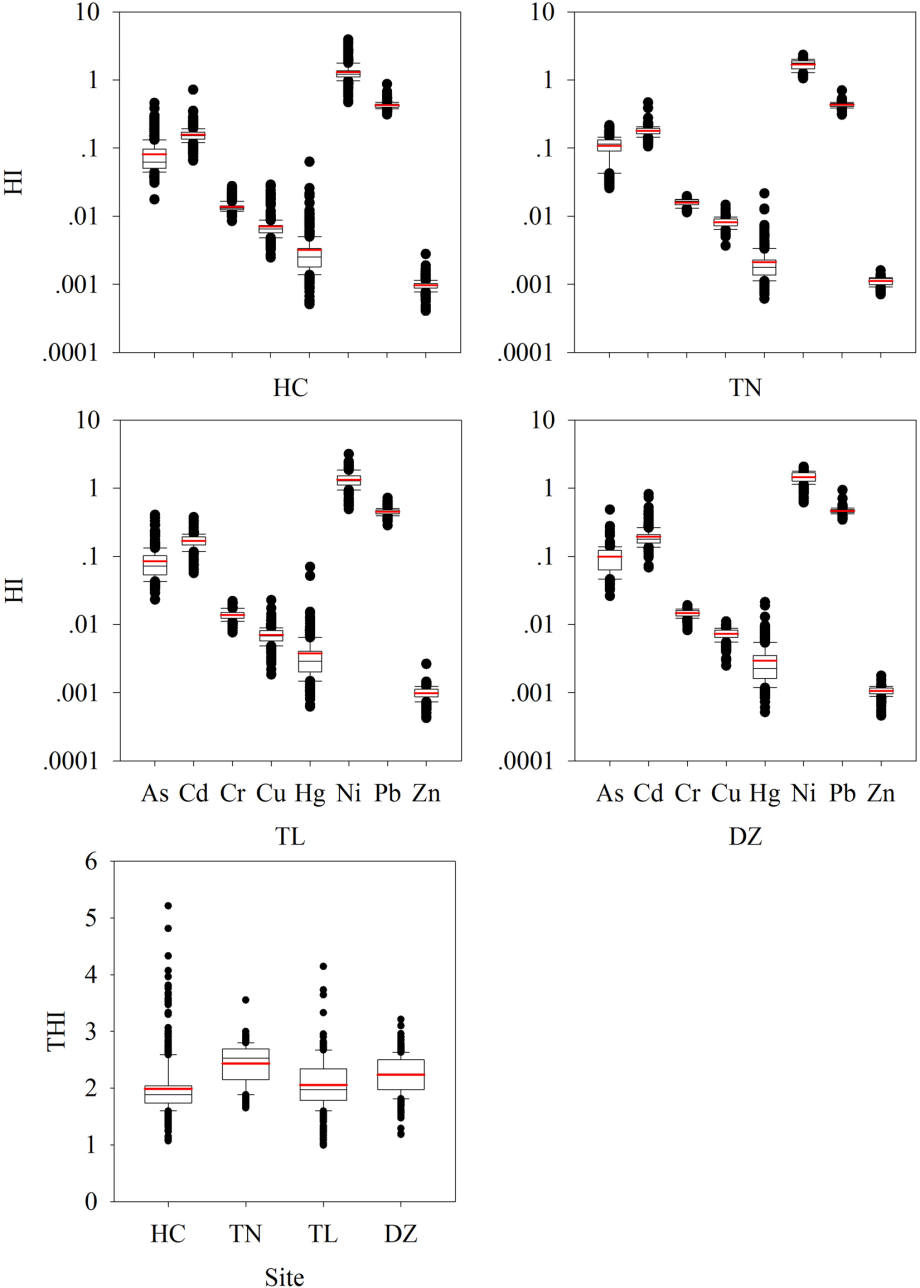
HI for children exposure to HMs in soils of each County (THI for each county showed significant differences, p < 0.001 by Mann-Whitney U text) (symbols for boxplot are similar to Fig. 2).

The results of non-carcinogenic risk of HM exposure in soils through non-dietary ingestion and inhalation, and dermal contact on adults and children are illustrated in Figs 4–6, and Tables S4 and S5 (differences are significant at p < 0.05). The HQ of ingestion and inhalation peaked for Pb and reached its minimal level for Cr irrespective of adults and children, while HQ of dermal showed different trends. HQ_oral_ in each County was found in the order of Pb > As > Ni > Cu ~ Hg ~ Cd ~ Zn > Cr for both adults and children, and HQ_dermal_ always followed the order of Ni > Cd ~ Pb > As > Cr > Cu > Hg > Zn regardless of Counties and ages (Fig. 4). In general, HI median and mean values of all HMs for adults were much lower than unity (Fig. 5), indicating that there is no significant non –carcinogenic risk. Compared with adults, children had higher values of non - carcinogenic risk, i.e., HI average of Ni was greater than 1 in all the Counties, demonstrating non – carcinogenic effect of soil HM on children in this area. As a whole, total HI from HMs was 5.5 times higher for children than adults (Figs 5 and 6). Ni showed highest HI values, followed by Pb, both for adults and children (Fig. 6). The HI values from non – carcinogenic risk for both adults and children decreased in the order of Ni > Pb > Cd > As > Cr > Cu > Hg > Zn (Figs 5 and 6). Further, total HI among Counties also represented significant differences (p < 0.001 by Mann-Whitney U text) with the following descending order of TN > DZ > TL > HC for both adults and children (Figs 5 and 6).

Carcinogenic risks of the HMs (Cd, Cr and Pb) and metalloid As are shown at mean, standard deviation (S.D.), the 5th, and 95th percentiles in Table 3. The CRs in different County decreased in the order Cr > As > Cd > Pb both for adults and children. The averaged carcinogenic risks posed by Cr, As, Cd and Pb in soils for adults were lower than those for children, resulting in 1.38 times higher combined CR for children with respect to adults (Table 3). It was found that all the samples showed CR far below the acceptable threshold value of 1.0E-04 established by USEPA, indicating no significant long-term health effects. Lifetime carcinogenic risk values for adults and children were 15.5 and 21.5 × 10^−6^, respectively (Table 3). The lifetime carcinogenic risks for both adults and children were thus within tolerable of acceptable risk (1.0E-06-1.0E-04).

**Table 3:** Carcinogenic risk (×10^−6^) for different exposure pathways for adult and child (full name for abbreviations please see Table 1).

		N	Adult						Child					
			Mean	S.D.	95% CI		Min.	Max.	Mean	S.D.	95% CI		Min.	Max.
					LB	UB					LB	UB		
As	HC	582	2.06	1.28	1.95	2.16	0.45	11.65	2.85	1.78	2.71	3.00	0.62	16.14
	TN	385	2.74	0.95	2.65	2.84	0.66	5.49	3.80	1.32	3.66	3.93	0.91	7.61
	TL	337	2.16	1.28	2.02	2.30	0.59	10.30	2.99	1.78	2.80	3.18	0.82	14.26
	DZ	360	2.50	1.10	2.39	2.62	0.67	12.32	3.47	1.53	3.31	3.63	0.92	17.06
	Total	1664	2.33	1.21	2.28	2.39	0.45	12.32	3.23	1.67	3.15	3.31	0.62	17.06
Cd	HC	582	1.57	0.40	1.54	1.60	0.66	7.25	2.17	0.56	2.12	2.21	0.91	10.00
	TN	385	1.80	0.30	1.77	1.84	1.07	4.72	2.49	0.42	2.45	2.53	1.47	6.51
	TL	337	1.69	0.41	1.64	1.73	0.57	3.79	2.33	0.56	2.27	2.39	0.79	5.22
	DZ	360	1.96	0.76	1.88	2.04	0.69	8.22	2.70	1.04	2.59	2.81	0.95	11.33
	Total	1664	1.73	0.51	1.71	1.76	0.57	8.22	2.39	0.70	2.36	2.42	0.79	11.33
Cr	HC	582	10.86	1.85	10.71	11.01	6.75	21.90	15.03	2.56	14.82	15.24	9.34	30.29
	TN	385	12.65	1.41	12.51	12.79	9.08	15.68	17.51	1.95	17.31	17.70	12.57	21.69
	TL	337	10.92	1.90	10.71	11.12	6.07	17.48	15.11	2.62	14.82	15.39	8.39	24.19
	DZ	360	11.60	1.41	11.45	11.74	6.54	15.10	16.05	1.95	15.84	16.25	9.04	20.90
	Total	1664	11.45	1.82	11.36	11.53	6.07	21.90	15.84	2.52	15.72	15.96	8.39	30.29
Pb	HC	580	0.0284	0.0031	0.0282	0.0287	0.02	0.06	0.0396	0.0043	0.0393	0.0400	0.03	0.08
	TN	385	0.0289	0.0024	0.0287	0.0292	0.02	0.05	0.0403	0.0033	0.0400	0.0407	0.03	0.07
	TL	337	0.0301	0.0034	0.0297	0.0304	0.02	0.05	0.0419	0.0047	0.0414	0.0424	0.03	0.07
	DZ	360	0.0310	0.0031	0.0307	0.0313	0.02	0.06	0.0432	0.0043	0.0428	0.0437	0.03	0.09
	Total	1662	0.0294	0.0032	0.0293	0.0296	0.02	0.06	0.0410	0.0044	0.0408	0.0412	0.03	0.09

We therefore concluded that children were more susceptible to the potential health regardless of the carcinogenic or non – carcinogenic risk. This finding was in good agreements with other studies^26–28^. Pan *et al*.^28^ reported children showed 7-fold higher HI for non – carcinogenic risk, and 1.7-fold higher CR than adults. Meanwhile, the HQ values of all HMs via ingestion for adults were three orders of magnitudes higher than those via inhalation, and a similar trend for children was also observed, with four-order of magnitudes higher HQ for children (data are not shown). Though past studies reported large differences between HI values via ingestion and dermal contact^18,27^, which was inconsistent with our findings, this was mainly contributable to different absorption factor^27–29^. Different dermal absorption factor (ca. 0006–0.35) (Table 4) for individual HM rather than similar absorption factor (ca. 0.001)^27^ was adopted in our calculations, resulting in much higher ABS and thus higher HQ_dermal_.

**Table 4:** Parameters for health risk assessment.

Description	Parameters	Value	Units	References
Ingestion rate of soil	IR_ing_	20 for adults and 50 for children	mg/day	^39^
Inhalation rate of soil	IR_inh_	16 for adults and 7.6 for children	m^3^/day	^39^
Skin area available for soil contact	SA	5700 for adults and 2800 for children	cm^2^	^30^
Soil-to-skin adherence factor	AF	2 × 10^−7^ for adults and 1 × 10^−6^ for children	kg/cm^2^/day	^39^
Dermal absorption factor (As)	ABS_As_	0.03		^31^
Dermal absorption factor (Cd)	ABS_Cd_	0.14		^31^
Dermal absorption factor (Cr)	ABS_Cr_	0.04		^31^
Dermal absorption factor (Cu)	ABS_Cu_	0.1		^31^
Dermal absorption factor (Hg)	ABS_Hg_	0.05		^31^
Dermal absorption factor (Ni)	ABS_Ni_	0.35		^31^
Dermal absorption factor (Pb)	ABS_Pb_	0.006		^31^
Dermal absorption factor (Zn)	ABS_Zn_	0.02		^31^
Particle emission factor	PEF	1.36 × 10^9^	m^3^/kg	^30^
Exposure frequency	EF	350	day/year	
Exposure duration	ED	24 for adults and 6 for children	year	^39^
Body weight	BW	65 for adults and 29 for children	kg	^9^
Average time	AT	ED × 365	day	^9^
Life time expressed in day	LT	76.6 × 365	day	

*0.001 for absorption factor (ABS) of all the HMs was also widely used elsewhere.

The health risk assessment shows powerful capacity to distinguish the toxic chemical and various exposure pathways. However, this assessment has several inherent uncertainties in quantitative risk evaluation. Firstly, bioavailable or bioaccessible concentration rather than the total amounts of HMs can obtain more reliable risk assessments for eco-environment and human health, which suggests that total concentration of HMs potentially results in overestimation of the ADI and the resulting HI. Secondly, the widely used exposure parameters were from the USEPA, which may not be applicable in China. However, there is no exposure assessment guideline for human health risk assessment posed by HMs in soils. Thirdly, as mentioned above, there are large differences in ABS of HMs from USEPA and Canada^30,31^, nevertheless, they are both widely used. However, our study scored the eco-environmental and human health effects based on a high-spatial-resolution investigation, particularly, three exposure pathways and variable HMs leading to potential ecological and human health risks in our typical area are highlighted.

## Conclusions

Multiple modern indices were used to completely assess eo-environmental and human health risks based on a high-spatial-resolution sampling in a typical area of the upper Yangtze Basin, a rapidly developing area in China. Averages and 95% confidence interval for mean of HMs were below the grade II criterion of the Chinese Environmental Quality standards for soils, while Cd, As and Hg were much higher than local background values. EF suggested overall moderate enrichments of Cd and Se, as well as significant enrichments of As, Cd and Hg in some sites as a consequence of anthropogenic inputs. Thus, soils were uncontaminated to moderately contaminated by Cd and Se, and soils were uncontaminated by other HMs. Potential ecological risk assessment showed significant differences in RI among counties as follows: DZ (184.9 ± 57.6; Mean ± S.D.) ≈ TL (182.0 ± 90.8) > TN (165.6 ± 36.9) ≈ HC (165.4 ± 71.3), demonstrating moderate risk of HMs. However, we needed to highlight that several sites were moderately to heavily contaminated with As, Cd and Hg by Igeo, resulting in that these sites were categorized as “considerable risk”, or “high risk”. Moreover, regardless of the carcinogenic or non – carcinogenic risk, children were found to be more susceptible to the potential health risk; children were therefore likely under a higher health risk than adults. There were no significant carcinogenic and non – carcinogenic risks for adults, while children showed significant non – carcinogenic effect. Though no serious pollution posed by HMs was found in this area, large variability of soil HMs in China’s urbans and those sites with high pollution level in our study area should be highlighted, and government needs to make efforts to reduce the effects of soil HMs on human health and eco - environmental risks.

## Materials and Methods

### Study area

The study was performed in the four typical counties (i.e., Huchuan, Tongnan, Tongliang and Dazu) of the Chongqing in the upper Yangtze Basin (Fig. 1). Chongqing (105°11′-110^0^11′E; 28^0^10′-32^0^13′N), a municipality directly under the jurisdiction of central government, is situated in the southwest part of China (Fig. 1). Chongqing has a total population of approximately 31 million and an area of 82,400 km^2^. Rapid industrialization and expansion of the population are increasing industrial and municipal wastewater discharges in this region. Hechuan (29.51′-30.22′N, 105.58′-106.40′E), Tongnan (29°47′-30°26′N, 105°31′-106°00′E), Tongliang (29°31′-30°5′N, 105°46′-106°16′E) and Dazu (29°23′-29°52′N, 105°28′-106°2′E) show a population of 1.56, 0.94, 0.84, and 0.85 million, and spans over 1108, 990, 1075 and 1009 km^2^, respectively. The area has a humid subtropical continental monsoonal climate with an average annual mean temperature and rainfall of 17–18 °C and 990–1108 mm, respectively.

The four counties are important agricultural bases, mainly producing vegetables, grain, fruits, and poultry, as well as aquaculture. The dominant land is cultivated land with a proportion of 52% for Hechuan, 74% for Tongnan, 62.7% for Tongliang, and 64.8% for Dazu, respectively. The geological strata exposed are mainly Jurassic purple sand and shale. Soils are typically purple soil and paddy soil.

### Sampling and analysis

The soil sampling method was similar to that described elsewhere^32^. In brief, a total of 6656 surface (0–20 cm) soil samples from across four Counties were collected in 2009 using a gridded sampling design with a grid spacing of approximately 1 km to represent the whole area. Generally, an optimum sampling density of one sample every km^2^ was taken and four subsamples were pooled together for one composite sample (one sample per 4 km^2^). As a result, a total of 1664 surface soil samples (582, 385, 337 and 360 samples for Hechuan, Tongnan, Tongliang and Dazu, respectively) were obtained for laboratory measurements.

All samples were air-dried, sieved through a 20-mesh nylon sieve after clearing visible debris, pebbles and stones, and then milled with an agate grinder until fine particles (< 200 μm) were obtained for analyses. Each dried sample was kept in brown glass bottles before analysis. Samples were analyzed in a special laboratory of Ministry of land and resources of China as follows. Approximately 1 g of milled soil sample was digested using a mixed strong acid (HCl, HNO_3_ and HClO_4_) pseudo-total digestion method^33^. Concentrations of Cd, Cu, Ni, Co, Mn, Mo, Sc and Sn were determined using inductively coupled plasma mass spectrometry (ICP-MS; Thermo XSeries II, USA) or inductively coupled plasma optical emission spectrometry (ICP-OES; Thermo ICAP 6300, USA). Concentrations of As, Hg, Se and Sb were measured using atomic fluorescence spectrometry (AFS). 5 g fine soil samples were used for measurements of Cr, Pb, Zn, Sr, Ti and major elements (Si, Al and Fe) by X-ray fluorescence spectrometry (XRF; primus II, Japan). Soil pH was measured in water - soil suspension (ration of mass weight of soil and deionized water is 1:2.5). The glass and plastic ware was soaked overnight using a HNO_3_ solution (10%, v/v) and then rinsed thoroughly with ultrapure deionized water. Ultra-pure acids were used for digestion and other reagents were of analytical grade.

Quality control included blind duplicates and insertion of standard reference materials (RM). There are 5% of the total samples, and 12 reference samples in 500 determined for quality assurance programme. Duplicate and reference samples were measured in parallel to each batch of samples using identical procedures. Data for analytical quality assurance including limit of detection (LOD), measurements for reference materials (RM) were supplied in the Table S6 (Supplementary material), and show high quality of our HMs concentrations in soils.

### Evaluation of environmental risk

#### Index of geoaccumulation

Geoaccumulation index (I_geo_), introduced by Muller (1969), has been widely used to assess the contamination levels of HMs in soils^34^. It can be computed using the following equation (1).

1 $${{\rm{I}}}_{{\rm{geo}}}={\mathrm{log}}_{2}(\frac{{{\rm{C}}}_{{\rm{n}}}}{1.5{{\rm{B}}}_{{\rm{n}}}})$$

where C_n_ is the measured concentration of every element, B_n_ is the local geochemical background value of HMs. The background geochemical compositions of HMs are from Chongqing (see Table 2). The constant 1.5 is adopted because of natural fluctuation of baseline data. I_geo_ shows 7 classes as: uncontaminated (I_geo_ ≤ 0), uncontaminated to moderate contaminated (0 < I_geo_ ≤ 1), moderate contaminated (1 < I_geo_ ≤ 2), moderately to heavily contaminated (2 < I_geo_ ≤ 3), heavily contaminated (3 < I_geo_ ≤ 4), heavily to extremely contaminated (4 < I_geo_ ≤ 5), and extremely contaminated (I_geo_ > 5). The summary statistics of I_geo_ of HMs are listed in Tables S1 and S2, as well as Fig. 2a.

### Enrichment factor

EF is employed to assess the degree of human effects on soil HMs, which can be expressed as follows^35,36^:

2 $${\rm{EF}}=\frac{{({{\rm{C}}}_{{\rm{i}}}/{{\rm{C}}}_{{\rm{ref}}})}_{{\rm{sample}}}}{{({{\rm{C}}}_{{\rm{i}}}/{{\rm{C}}}_{{\rm{ref}}})}_{{\rm{bakbround}}}}$$

where C_i_ is the concentration of the ith element, C_ref_ is the concentration of reference metal for normalization. Here, Al was chosen as reference because of its abundant content and stability in the crust. EF can be categorized to 5 levels: minimal enrichment (<2), moderate enrichment (2 ≤ EF < 5), significant enrichment (5 ≤ EF < 20), very high enrichment (20 ≤ EF < 40), and extremely enrichment (EF ≥ 40). The summary statistics of EF of HMs are illustrated in Tables S1 and S3, as well as Fig. 2b.

### Evaluation of potential ecological risk

Hakanson RI, a comprehensive method combining all of HMs with their toxicological effects, was adopted for evaluations of potential ecological risks posed by HM pollution^37^. This method has been widely used to evaluate ecological risks caused by HMs in soils^28,38^, and can be calculated using the following equations:

3 $${\rm{RI}}=\sum _{{\rm{i}}=1}^{{\rm{n}}}{{\rm{E}}}_{{\rm{r}}}^{{\rm{i}}}$$

4 $${{\rm{E}}}_{{\rm{r}}}^{{\rm{i}}}={{\rm{T}}}_{{\rm{r}}}^{{\rm{i}}}\times \frac{{{\rm{C}}}^{{\rm{i}}}}{{{\rm{C}}}_{{\rm{r}}}^{{\rm{i}}}}$$

where ${{\rm{T}}}_{{\rm{r}}}^{{\rm{i}}}$ is the toxic response factor for a given substance, they are 40, 30, 10, 5, 5, 5, 2, and 1 for Hg, Cd, As, Cu, Ni, Pb, Cr and Zn, respectively. ${{\rm{C}}}^{{\rm{i}}}$ is the measured metal concentration. ${C}_{r}^{i}$ is referred to the background value of HM in soils of Chongqing, China (Table 2). ${{\rm{E}}}_{{\rm{r}}}^{{\rm{i}}}$ is individual potential ecological risk factor, and RI is a composite index that represents potential ecological risk of total HMs in soils. n is the total numbers of the determined HMs. RI is generally defined as four grades: low risk (RI ≤ 150), moderate risk (150 < RI ≤ 300), considerable risk (300 < RI ≤ 600) and high risk (RI > 600)^28,38^.

### Health risk assessment

#### Exposure assessment

Human health risk assessment is a popular method for quantifying the nature and probability of adverse health effects on humans who are exposed to certain HMs^13,24^. For soils, in general, individuals are exposed to soil contaminants through three major pathways of ingestion, dermal absorption and inhalation. The former two are the main exposure pathways^28,39^. To evaluate risks of human exposure to HMs on both adults and children, the average daily intake (ADI) (mg/kg/d) is introduced^35,40^.

5 $${{\rm{ADI}}}_{{\rm{ing}}}=\frac{{{\rm{C}}}_{{\rm{i}}}\times {{\rm{IR}}}_{{\rm{ing}}}\times {\rm{EF}}\times {\rm{ED}}}{{\rm{BW}}\times {\rm{AT}}}$$

6 $${{\rm{ADI}}}_{{\rm{der}}}=\frac{{{\rm{C}}}_{{\rm{i}}}\times {\rm{SA}}\times {\rm{AF}}\times {\rm{ABS}}\times {\rm{EF}}\times {\rm{ED}}}{{\rm{BW}}\times {\rm{AT}}}$$

7 $${{\rm{ADI}}}_{{\rm{inh}}}=\frac{{{\rm{C}}}_{{\rm{i}}}\times {{\rm{IR}}}_{{\rm{inh}}}\times {\rm{EF}}\times {\rm{ED}}}{{\rm{PEF}}\times {\rm{BW}}\times {\rm{AT}}}$$

where ADI_ing_, ADI_der_ and ADI_inh_ (mg/kg/d) respectively represent the ADI of HMs through ingestion, dermal contact and inhalation (mg/kg/d); C_i_ is the HM concentration in the soil; IR_ing_ (mg/d) and IR_inh_ (m^3^/d) are ingestion and inhalation rates of soil; EF is the exposure frequency (day/year); ED is the exposure duration (year); SA is the posed surface area of skin (cm^2^); AF is the adherence factor (kg/m^2^/day); ABS is the dermal absorption factor; PEF is the particle emission factor (m^3^/kg); BW is the body weight (kg); AT is the average time (day). The exposure factors used for calculations of ADI are listed in Table 4.

### Non-carcinogenic risk assessment

The hazard quotient (HQ) is used for evaluation of non-carcinogenic risk, which is calculated by dividing ADD by the reference dose (RfD) for a specific substance^30^. HQ is defined using equation (8).

8 $${\rm{HQ}}=\frac{{\rm{ADI}}}{{\rm{RfD}}}$$

where RfD (mg/kg/day) refers to the reference dose of a given HM. RfD values for different HM are listed in Table 5.

**Table 5:** Reference dose (RfD) values of heavy metals^29^.

	ABS_GI_	RfD_o_	RfD_ABS_
		mg/kg/day	mg/kg/day
As	1	3.00E-04	3.00E-04
Cd	0.025	1.00E-03	2.50E-05
Cr	0.013	1.50E + 00	1.95E-02
Cu	1	4.00E-02	4.00E-02
Hg	1	1.60E-04	1.60E-04
Ni	0.04	2.00E-02	8.00E-04
Pb	1	1.40E-04	1.40E-04
Zn	1	3.00E-01	3.00E-01

Reference doses are not available for dermal absorption exposure to contaminants, RfD_ABS_ is calculated by using RfD_ABS_ = RfD_o_ × ABS_GI_

where RfD_ABS_ is the dermally adjusted reference dose (mg/kg/day), RfDo is the oral reference dose (mg/kg/day), and ABS_GI_ is the gastrointestinal absorption factor (unitless)^30^.

Hazard index (HI) that is defined as the sum of HQ is applied to assess the overall potential non-carcinogenic posed by measured HMs. Equation of HI is expressed as follows:

9 $${\rm{HI}}={\sum }^{}H{Q}_{i}={\sum }^{}\frac{AD{I}_{i}}{Rf{D}_{i}}$$

If the HI is larger than unity, non-carcinogenic effects of HMs to exposed individual may occur, while there is little chance of adverse health effects of HMs to human health if HI is below one.

### Carcinogenic risk assessment

The carcinogenic risk (CR) can be calculated as individual lifetime cancer risk by multiplying lifetime average daily doses (LADD) with the cancer slope factor (CSF, unit in per mg/kg/day).

10 $${\rm{CR}}={\rm{LADD}}\times {\rm{CSF}}$$

Several chemical such as As, Cd, Cr and Pb of our determined HMs can result in carcinogenic risks, thus, the total cancer risk (lifetime carcinogenic risk) is expressed as the summation of the individual CR^39^. Calculations of LADD are based on Equations 5–7 using LT instead of AT. The CSF values of As, Cd, Cr and Pb are 1.5, 6.3, 0.5, and 0.0085 per (mg/kg/day)^−1^, respectively^27,41^. The acceptable threshold value of the CR is 1.0E-4, CR values exceeding 1.0E-4 indicates potential of a lifetime carcinogenic risk^30^.

### Statistical analyses

Descriptive statistics including arithmetic mean, maximum, minimum, standard deviation, standard errors, and 95% confidence intervals (CI) are calculated and listed in Tables 1, 2 and S1. One sample Kolmogorov - Smirnov test was carried out to normality of the data distribution. Data that were not normally distributed were transformed by natural logarithm for statistical analyses. Analysis of variance (ANOVA) was performed to compare the differences of concentrations between counties. The statistical significance level was p < 0.05. The statistical processes were conducted using SigmaPlot 11 and SPSS 16.0 software.

## Electronic supplementary material

Supplementary Information
