# Supplementary material for: Assessment of soil heavy metals for eco-environment and human health in a rapidly urbanization area of the upper Yangtze Basin
Source: Sci Rep. 2018 Feb 19;8:3256. doi: 10.1038/s41598-018-21569-6 (PMC5818520; doi:10.1038/s41598-018-21569-6)
Supplement: Supplementary file 1 — Supplementary Information [file 41598_2018_21569_MOESM1_ESM.pdf]

SUPPORTING MATERIALS

Assessment of soil heavy metals for eco-environment and human health in a rapidly urbanization area of the upper Yangtze Basin

Zhongmin Jia[1,2], Siyue Li[3]*, Li Wang[1]

1. Key Laboratory of Eco-environments of the Three Gorges Reservoir Region, Ministry of Education, College of Life Science, Southwest University, Chongqing 400715, China

2. Southeast Sichuan Geological Group, Chongqing Bureau of Geology and Minerals Exploration, Chongqing 400038, China

3. Chongqing Institute of Green and Intelligent Technology, Chinese Academy of Sciences, Chongqing 400714, China

Table S1. Igeo and EF in the 4 counties of the upper Yangtze Basin.

			Igeo							EF						
		N	Mean	S.D.	S.E.	95% CI		Min.	Max.	Mean	S.D.	S.E.	95%CI		Min.	Max.
						LB	UB						LB	UB		
As	HC	582	-0.64	0.68	0.03	-0.69	-0.58	-2.64	2.05	1.11	0.68	0.03	1.05	1.16	0.24	5.32
	TN	385	-0.16	0.64	0.03	-0.22	-0.09	-2.1	0.96	1.47	0.52	0.03	1.41	1.52	0.35	3.16
	TL	337	-0.56	0.69	0.04	-0.64	-0.49	-2.26	1.87	1.17	0.69	0.04	1.1	1.25	0.3	5.21
	DZ	360	-0.3	0.63	0.03	-0.37	-0.24	-2.08	2.13	1.35	0.63	0.03	1.28	1.41	0.34	7
	Total	1664	-0.44	0.69	0.02	-0.47	-0.41	-2.64	2.13	1.26	0.65	0.02	1.22	1.29	0.24	7
Cd	HC	582	0.83	0.31	0.01	0.8	0.85	-0.39	3.07	2.75	0.66	0.03	2.7	2.81	1.16	10.68
	TN	385	1.05	0.22	0.01	1.02	1.07	0.31	2.45	3.15	0.53	0.03	3.09	3.2	1.86	7.95
	TL	337	0.92	0.38	0.02	0.88	0.96	-0.6	2.13	2.97	0.68	0.04	2.9	3.05	1.14	6.49
	DZ	360	1.11	0.43	0.02	1.07	1.16	-0.32	3.25	3.42	1.29	0.07	3.29	3.56	1.41	13.99
	Total	1664	0.96	0.36	0.01	0.94	0.98	-0.6	3.25	3.03	0.86	0.02	2.99	3.08	1.14	13.99
Cr	HC	582	-0.76	0.22	0.01	-0.78	-0.74	-1.43	0.27	0.9	0.13	0.01	0.89	0.91	0.66	1.72
	TN	385	-0.53	0.17	0.01	-0.55	-0.52	-1	-0.21	1.05	0.1	0.01	1.04	1.06	0.76	1.25
	TL	337	-0.76	0.25	0.01	-0.79	-0.73	-1.58	-0.06	0.91	0.13	0.01	0.9	0.93	0.64	1.54
	DZ	360	-0.66	0.18	0.01	-0.68	-0.64	-1.48	-0.27	0.96	0.1	0.01	0.95	0.97	0.62	1.22
	Total	1664	-0.69	0.23	0.01	-0.7	-0.68	-1.58	0.27	0.95	0.13	0	0.94	0.96	0.62	1.72
Cu	HC	582	-0.68	0.46	0.02	-0.71	-0.64	-2.1	1.45	1	0.43	0.02	0.97	1.04	0.42	4.05
	TN	385	-0.39	0.25	0.01	-0.42	-0.37	-1.52	0.47	1.16	0.17	0.01	1.14	1.18	0.51	2.01
	TL	337	-0.66	0.42	0.02	-0.7	-0.61	-2.52	1.09	1	0.26	0.01	0.97	1.03	0.37	3.07
	DZ	360	-0.58	0.28	0.01	-0.61	-0.55	-2.08	0.05	1.03	0.17	0.01	1.01	1.04	0.41	1.53
	Total	1664	-0.59	0.39	0.01	-0.61	-0.57	-2.52	1.45	1.04	0.31	0.01	1.03	1.06	0.37	4.05
Hg	HC	581	-0.44	0.81	0.03	-0.5	-0.37	-2.79	4.15	1.38	1.55	0.06	1.26	1.51	0.2	26.72

	TN	385	-0.93	0.66	0.03	-0.99	-0.86	-2.52	2.62	0.91	0.73	0.04	0.84	0.99	0.26	9.16
	TL	337	-0.26	0.88	0.05	-0.35	-0.16	-2.49	4.31	1.63	2.15	0.12	1.4	1.86	0.25	30.08
	DZ	360	-0.54	0.84	0.04	-0.62	-0.45	-2.76	2.59	1.27	0.97	0.05	1.17	1.37	0.22	8.47
	Total	1663	-0.54	0.83	0.02	-0.58	-0.5	-2.79	4.31	1.3	1.47	0.04	1.23	1.37	0.2	30.08
Ni	HC	582	-0.63	0.37	0.02	-0.66	-0.6	-2.05	1.01	1.01	0.28	0.01	0.98	1.03	0.46	2.71
	TN	385	-0.23	0.25	0.01	-0.25	-0.2	-0.88	0.26	1.3	0.19	0.01	1.28	1.32	0.85	1.74
	TL	337	-0.6	0.4	0.02	-0.65	-0.56	-1.99	0.7	1.03	0.25	0.01	1.01	1.06	0.52	2.34
	DZ	360	-0.45	0.28	0.01	-0.48	-0.42	-1.66	0.08	1.12	0.18	0.01	1.1	1.14	0.55	1.54
	Total	1664	-0.49	0.37	0.01	-0.51	-0.48	-2.05	1.01	1.11	0.26	0.01	1.09	1.12	0.46	2.71
Pb	HC	580	-0.54	0.15	0.01	-0.55	-0.53	-0.98	0.5	1.05	0.11	0	1.04	1.05	0.74	2.04
	TN	385	-0.51	0.12	0.01	-0.52	-0.5	-0.99	0.19	1.06	0.08	0	1.05	1.07	0.86	1.69
	TL	337	-0.46	0.16	0.01	-0.48	-0.44	-1.11	0.23	1.11	0.12	0.01	1.1	1.13	0.82	1.98
	DZ	360	-0.41	0.13	0.01	-0.43	-0.4	-0.83	0.6	1.14	0.13	0.01	1.13	1.15	0.91	2.81
	Total	1662	-0.49	0.15	0	-0.5	-0.48	-1.11	0.6	1.08	0.12	0	1.08	1.09	0.74	2.81
Zn	HC	582	-0.56	0.26	0.01	-0.58	-0.54	-1.78	0.99	1.04	0.18	0.01	1.03	1.06	0.49	2.95
	TN	385	-0.34	0.18	0.01	-0.36	-0.33	-0.97	0.2	1.19	0.12	0.01	1.18	1.2	0.76	1.67
	TL	337	-0.53	0.32	0.02	-0.57	-0.5	-1.72	0.92	1.07	0.2	0.01	1.05	1.09	0.57	2.92
	DZ	360	-0.41	0.22	0.01	-0.43	-0.39	-1.6	0.34	1.14	0.14	0.01	1.13	1.16	0.57	1.83
	Total	1664	-0.47	0.27	0.01	-0.49	-0.46	-1.78	0.99	1.1	0.18	0	1.1	1.11	0.49	2.95
Co	HC	582	-0.64	0.35	0.01	-0.67	-0.61	-2.26	1.09	1	0.29	0.01	0.97	1.02	0.33	3.05
	TN	385	-0.53	0.18	0.01	-0.54	-0.51	-1.15	-0.2	1.05	0.1	0.01	1.04	1.06	0.73	1.39
	TL	337	-0.72	0.36	0.02	-0.76	-0.68	-2.29	0.68	0.94	0.2	0.01	0.92	0.97	0.38	2.31
	DZ	360	-0.58	0.21	0.01	-0.6	-0.56	-1.81	-0.11	1.02	0.13	0.01	1	1.03	0.54	1.41
	Total	1664	-0.62	0.3	0.01	-0.63	-0.6	-2.29	1.09	1	0.21	0.01	0.99	1.01	0.33	3.05
Mn	HC	582	-0.77	0.4	0.02	-0.8	-0.73	-3.17	0.64	0.92	0.23	0.01	0.9	0.94	0.16	2.2
	TN	385	-0.58	0.24	0.01	-0.61	-0.56	-1.56	0.33	1.02	0.14	0.01	1	1.03	0.51	1.82

	TL	337	-0.86	0.5	0.03	-0.91	-0.81	-2.94	0.31	0.88	0.22	0.01	0.85	0.9	0.2	1.67
	DZ	360	-0.78	0.39	0.02	-0.82	-0.74	-2.57	-0.17	0.91	0.2	0.01	0.89	0.93	0.29	1.29
	Total	1664	-0.75	0.4	0.01	-0.77	-0.73	-3.17	0.64	0.93	0.21	0.01	0.92	0.94	0.16	2.2
Mo	HC	582	-0.46	0.54	0.02	-0.5	-0.41	-1.32	1.7	1.2	0.54	0.02	1.15	1.24	0.57	4.19
	TN	385	0.27	0.43	0.02	0.23	0.32	-0.94	1.72	1.89	0.51	0.03	1.84	1.94	0.8	4.77
	TL	337	-0.32	0.5	0.03	-0.38	-0.27	-1.23	1.39	1.3	0.49	0.03	1.25	1.35	0.65	3.8
	DZ	360	-0.04	0.48	0.03	-0.09	0.01	-1.18	1.9	1.56	0.53	0.03	1.5	1.61	0.66	5.45
	Total	1664	-0.17	0.57	0.01	-0.2	-0.14	-1.32	1.9	1.46	0.59	0.01	1.43	1.48	0.57	5.45
Ti	HC	582	-0.73	0.31	0.01	-0.75	-0.7	-1.04	1.15	0.94	0.29	0.01	0.92	0.96	0.71	3.28
	TN	385	-0.77	0.07	0	-0.78	-0.76	-1.03	-0.48	0.88	0.06	0	0.88	0.89	0.75	1.28
	TL	337	-0.78	0.19	0.01	-0.8	-0.76	-1.2	0.44	0.9	0.14	0.01	0.88	0.91	0.73	1.97
	DZ	360	-0.76	0.1	0.01	-0.77	-0.74	-1.07	-0.32	0.9	0.08	0	0.89	0.91	0.72	1.45
	Total	1664	-0.76	0.21	0.01	-0.77	-0.75	-1.2	1.15	0.91	0.19	0	0.9	0.92	0.71	3.28
Sb	HC	582	-0.93	0.46	0.02	-0.97	-0.89	-2.19	2.74	0.85	0.46	0.02	0.81	0.89	0.33	9.46
	TN	385	-0.72	0.35	0.02	-0.76	-0.69	-3.11	0.38	0.94	0.2	0.01	0.92	0.96	0.17	1.89
	TL	337	-0.85	0.46	0.03	-0.9	-0.8	-2.47	2.78	0.91	0.58	0.03	0.85	0.97	0.28	10.2
	DZ	360	-0.74	0.32	0.02	-0.78	-0.71	-1.56	1.28	0.93	0.26	0.01	0.9	0.96	0.53	3.63
	Total	1664	-0.82	0.42	0.01	-0.84	-0.8	-3.11	2.78	0.9	0.41	0.01	0.88	0.92	0.17	10.2
Sc	HC	582	-0.78	0.28	0.01	-0.8	-0.75	-1.58	0.73	0.9	0.19	0.01	0.88	0.91	0.53	2.2
	TN	385	-0.63	0.25	0.01	-0.66	-0.61	-1.39	-0.21	0.98	0.14	0.01	0.97	1	0.58	1.26
	TL	337	-0.79	0.26	0.01	-0.81	-0.76	-1.66	0.22	0.9	0.14	0.01	0.88	0.91	0.59	1.68
	DZ	360	-0.71	0.21	0.01	-0.73	-0.69	-1.42	-0.31	0.93	0.12	0.01	0.92	0.94	0.64	1.18
	Total	1664	-0.73	0.26	0.01	-0.74	-0.72	-1.66	0.73	0.92	0.16	0	0.92	0.93	0.53	2.2
Se	HC	582	0.51	0.52	0.02	0.46	0.55	-0.57	3.13	2.33	1.18	0.05	2.23	2.43	0.99	12.53
	TN	385	0.21	0.35	0.02	0.18	0.25	-0.61	2.77	1.82	0.73	0.04	1.74	1.89	0.96	9.9
	TL	337	0.58	0.48	0.03	0.52	0.63	-0.71	2.27	2.45	1.1	0.06	2.33	2.57	0.93	8.5

	DZ	360	0.47	0.35	0.02	0.44	0.51	-0.33	1.96	2.18	0.72	0.04	2.11	2.26	1.18	6.1
	Total	1664	0.44	0.46	0.01	0.42	0.47	-0.71	3.13	2.2	1.01	0.02	2.15	2.25	0.93	12.53
Sn	HC	582	-0.72	0.22	0.01	-0.73	-0.7	-1.15	1.05	0.93	0.17	0.01	0.92	0.95	0.68	2.92
	TN	385	-0.74	0.22	0.01	-0.76	-0.71	-1.37	0.55	0.91	0.15	0.01	0.9	0.93	0.59	2.17
	TL	337	-0.73	0.2	0.01	-0.75	-0.71	-1.37	-0.29	0.93	0.14	0.01	0.92	0.95	0.54	1.44
	DZ	360	-0.66	0.2	0.01	-0.68	-0.64	-1.15	-0.05	0.96	0.14	0.01	0.95	0.98	0.72	1.48
	Total	1664	-0.71	0.21	0.01	-0.72	-0.7	-1.37	1.05	0.94	0.15	0	0.93	0.94	0.54	2.92

Table S2. Number of Igeo distribution based on Igeo classification.

	As	Cd	Cr	Cu	Hg	Ni	Pb	Zn	Co	Mn	Mo	Ti	Sb	Sc	Se	Sn
**HC**																
≤0	478	9	576	552	454	538	577	563	554	566	468	557	560	568	72	578
0-1	86	429	6	21	98	43	3	19	26	16	105	24	21	14	427	3
1-2	17	142		9	20	1			2		9	1			72	1
2-3	1	1			8								1		9	
3-4		1													2	
4-5					1											
≥5																
**TN**																
≤0	169		385	381	355	322	384	382	385	384	91	385	384	385	77	384
0-1	216	138		4	25	63	1	3		1	289		1		302	1
1-2		245			4						5				4	
2-3		2			1										2	
3-4																
4-5																
≥5																
**TL**																
≤0	278	11	337	327	231	324	333	335	332	334	252	333	325	334	23	337
0-1	49	184		9	77	13	4	2	5	3	82	4	11	3	256	
1-2	10	141		1	25						3				56	
2-3		1			2								1		2	
3-4					1											
4-5					1											
≥5																
**DZ**																
≤0	223	2	360	359	273	357	357	358	360	360	165	360	355	360	20	360
0-1	132	148		1	71	3	3	2			191		4		311	
1-2	4	194			14						4		1		29	
2-3	1	14			2											
3-4		2														
4-5																
≥5																

HC-Hechuan; TN-Tongnan, TL-Tongliang, DZ-Dazu

Table S3. Number of EF distribution based on classification

	As	Cd	Cr	Cu	Hg	Ni	Pb	Zn	Co	Mn	Mo	Ti	Sb	Sc	Se	Sn
**HC**																
≤2	536	34	582	559	510	570	579	580	569	577	533	568	578	580	278	581
2-5	45	543		23	59	12	1	2	13	5	49	14	3	2	283	1
5-20	1	5			11								1		21	
20-40					1											
>40																
**TN**																
≤2	337	2	379	378	360	379	379	379	379	379	204	379	379	379	301	378
2-5	42	375		1	16						175				75	1
5-20		2			3										3	
20-40																
>40																
**TL**																
≤2	309	21	337	334	269	336	337	336	335	337	309	337	335	337	143	337
2-5	27	311		3	60	1		1	2		28		1		178	
5-20	1	5			6								1		16	
20-40					2											
>40																
**DZ**																
≤2	319	11	337	337	285	337	336	337	337	337	289	337	336	337	157	337
2-5	17	300			49		1				47		1		173	
5-20	1	26			3						1				7	
20-40																
>40																

Table S4. Statistics of hazard quotient (HQ), and cumulative hazard index (HI) for non-carcinogenic health risk from HMs in soils (a-adults, b-children).

(a) Adults

	HQoral					HQdermal					HI
	Mean	Median	Std Dev	Max	Min	Mean	Median	Std Dev	Max	Min	
HC											
As	5.39E-03	4.13E-03	3.36E-03	3.05E-02	1.18E-03	9.21E-03	7.06E-03	5.75E-03	5.21E-02	2.02E-03	1.46E-02
Cd	8.87E-05	8.65E-05	2.28E-05	4.09E-04	3.72E-05	2.83E-02	2.76E-02	7.28E-03	1.31E-01	1.19E-02	2.84E-02
Cr	1.41E-05	1.35E-05	2.40E-06	2.84E-05	8.76E-06	2.47E-03	2.37E-03	4.21E-04	4.98E-03	1.54E-03	2.48E-03
Cu	1.92E-04	1.75E-04	8.73E-05	7.86E-04	6.72E-05	1.09E-03	9.96E-04	4.97E-04	4.48E-03	3.83E-04	1.28E-03
Hg	1.51E-04	1.18E-04	1.70E-04	2.96E-03	2.40E-05	4.30E-04	3.36E-04	4.83E-04	8.42E-03	6.83E-05	5.81E-04
Ni	4.74E-04	4.44E-04	1.46E-04	1.42E-03	1.71E-04	2.36E-01	2.21E-01	7.27E-02	7.09E-01	8.53E-02	2.36E-01
Pb	5.68E-02	5.63E-02	6.20E-03	1.16E-01	4.18E-02	1.94E-02	1.92E-02	2.12E-03	3.96E-02	1.43E-02	7.62E-02
Zn	8.15E-05	8.07E-05	1.57E-05	2.35E-04	3.44E-05	9.28E-05	9.19E-05	1.79E-05	2.67E-04	3.92E-05	1.74E-04
TN											
As	7.17E-03	7.65E-03	2.49E-03	1.44E-02	1.72E-03	1.23E-02	1.31E-02	4.25E-03	2.46E-02	2.94E-03	1.95E-02
Cd	1.02E-04	1.02E-04	1.71E-05	2.66E-04	6.02E-05	3.25E-02	3.27E-02	5.46E-03	8.49E-02	1.92E-02	3.26E-02
Cr	1.64E-05	1.70E-05	1.83E-06	2.04E-05	1.18E-05	2.88E-03	2.98E-03	3.20E-04	3.57E-03	2.07E-03	2.90E-03
Cu	2.22E-04	2.27E-04	3.62E-05	3.99E-04	1.00E-04	1.26E-03	1.29E-03	2.06E-04	2.27E-03	5.72E-04	1.48E-03
Hg	9.98E-05	8.30E-05	7.69E-05	1.02E-03	2.90E-05	2.84E-04	2.36E-04	2.19E-04	2.90E-03	8.25E-05	3.84E-04
Ni	6.14E-04	6.42E-04	1.02E-04	8.47E-04	3.84E-04	3.06E-01	3.20E-01	5.06E-02	4.22E-01	1.91E-01	3.07E-01
Pb	5.78E-02	5.80E-02	4.77E-03	9.36E-02	4.13E-02	1.98E-02	1.98E-02	1.63E-03	3.20E-02	1.41E-02	7.76E-02
Zn	9.38E-05	9.69E-05	1.14E-05	1.35E-04	6.01E-05	1.07E-04	1.10E-04	1.30E-05	1.54E-04	6.85E-05	2.01E-04
TL											
As	5.66E-03	4.82E-03	3.36E-03	2.70E-02	1.54E-03	9.67E-03	8.24E-03	5.74E-03	4.61E-02	2.64E-03	1.53E-02

Cd	9.53E-05	9.48E-05	2.30E-05	2.14E-04	3.22E-05	3.04E-02	3.02E-02	7.34E-03	6.82E-02	1.03E-02	3.05E-02
Cr	1.42E-05	1.39E-05	2.46E-06	2.27E-05	7.87E-06	2.48E-03	2.43E-03	4.31E-04	3.98E-03	1.38E-03	2.49E-03
Cu	1.90E-04	1.84E-04	5.57E-05	6.14E-04	5.02E-05	1.08E-03	1.05E-03	3.17E-04	3.50E-03	2.86E-04	1.27E-03
Hg	1.77E-04	1.35E-04	2.36E-04	3.30E-03	2.95E-05	5.05E-04	3.84E-04	6.71E-04	9.39E-03	8.41E-05	6.82E-04
Ni	4.84E-04	4.63E-04	1.32E-04	1.15E-03	1.79E-04	2.41E-01	2.31E-01	6.60E-02	5.75E-01	8.90E-02	2.41E-01
Pb	6.01E-02	5.99E-02	6.72E-03	9.64E-02	3.82E-02	2.05E-02	2.05E-02	2.30E-03	3.29E-02	1.30E-02	8.06E-02
Zn	8.35E-05	8.36E-05	1.78E-05	2.23E-04	3.59E-05	9.51E-05	9.53E-05	2.03E-05	2.54E-04	4.09E-05	1.79E-04
DZ											
As	6.56E-03	6.73E-03	2.88E-03	3.22E-02	1.74E-03	1.12E-02	1.15E-02	4.93E-03	5.51E-02	2.98E-03	1.78E-02
Cd	1.11E-04	1.02E-04	4.27E-05	4.64E-04	3.90E-05	3.53E-02	3.24E-02	1.36E-02	1.48E-01	1.24E-02	3.54E-02
Cr	1.51E-05	1.53E-05	1.83E-06	1.96E-05	8.48E-06	2.64E-03	2.69E-03	3.21E-04	3.44E-03	1.49E-03	2.66E-03
Cu	1.96E-04	1.97E-04	3.50E-05	2.99E-04	6.79E-05	1.12E-03	1.12E-03	1.99E-04	1.70E-03	3.87E-04	1.32E-03
Hg	1.38E-04	1.07E-04	1.05E-04	9.98E-04	2.45E-05	3.94E-04	3.04E-04	2.98E-04	2.84E-03	6.99E-05	5.32E-04
Ni	5.28E-04	5.33E-04	9.51E-05	7.50E-04	2.24E-04	2.63E-01	2.66E-01	4.74E-02	3.74E-01	1.12E-01	2.64E-01
Pb	6.20E-02	6.19E-02	6.15E-03	1.25E-01	4.62E-02	2.12E-02	2.12E-02	2.10E-03	4.27E-02	1.58E-02	8.32E-02
Zn	8.97E-05	9.05E-05	1.29E-05	1.50E-04	3.91E-05	1.02E-04	1.03E-04	1.47E-05	1.71E-04	4.45E-05	1.92E-04

(b) Children

	HQoral					HQdermal					HI
	Mean	Median	Std Dev	Max	Min	Mean	Median	Std Dev	Max	Min	
HC											
As	3.02E-02	2.31E-02	1.88E-02	1.71E-01	6.61E-03	5.07E-02	3.89E-02	3.16E-02	2.87E-01	1.11E-02	8.09E-02
Cd	4.97E-04	4.84E-04	1.28E-04	2.29E-03	2.08E-04	1.56E-01	1.52E-01	4.01E-02	7.19E-01	6.53E-02	1.56E-01
Cr	7.90E-05	7.58E-05	1.35E-05	1.59E-04	4.91E-05	1.36E-02	1.31E-02	2.32E-03	2.74E-02	8.45E-03	1.37E-02
Cu	1.07E-03	9.80E-04	4.89E-04	4.40E-03	3.76E-04	6.01E-03	5.49E-03	2.74E-03	2.47E-02	2.11E-03	7.08E-03

Hg	8.46E-04	6.61E-04	9.50E-04	1.66E-02	1.34E-04	2.37E-03	1.85E-03	2.66E-03	4.64E-02	3.76E-04	3.22E-03
Ni	2.66E-03	2.49E-03	8.17E-04	7.97E-03	9.59E-04	1.30E+00	1.22E+00	4.00E-01	3.90E+00	4.70E-01	1.30E+00
Pb	3.18E-01	3.15E-01	3.47E-02	6.50E-01	2.34E-01	1.07E-01	1.06E-01	1.17E-02	2.18E-01	7.86E-02	4.25E-01
Zn	4.56E-04	4.52E-04	8.79E-05	1.31E-03	1.93E-04	5.11E-04	5.06E-04	9.84E-05	1.47E-03	2.16E-04	9.67E-04
TN											
As	4.02E-02	4.28E-02	1.39E-02	8.05E-02	9.65E-03	6.75E-02	7.19E-02	2.34E-02	1.35E-01	1.62E-02	1.08E-01
Cd	5.71E-04	5.74E-04	9.58E-05	1.49E-03	3.37E-04	1.79E-01	1.80E-01	3.00E-02	4.68E-01	1.06E-01	1.80E-01
Cr	9.20E-05	9.51E-05	1.02E-05	1.14E-04	6.60E-05	1.58E-02	1.64E-02	1.76E-03	1.96E-02	1.14E-02	1.59E-02
Cu	1.24E-03	1.27E-03	2.03E-04	2.23E-03	5.62E-04	6.96E-03	7.11E-03	1.14E-03	1.25E-02	3.15E-03	8.20E-03
Hg	5.59E-04	4.65E-04	4.31E-04	5.71E-03	1.62E-04	1.56E-03	1.30E-03	1.21E-03	1.60E-02	4.54E-04	2.12E-03
Ni	3.44E-03	3.60E-03	5.69E-04	4.75E-03	2.15E-03	1.68E+00	1.76E+00	2.79E-01	2.33E+00	1.05E+00	1.69E+00
Pb	3.24E-01	3.25E-01	2.67E-02	5.24E-01	2.31E-01	1.09E-01	1.09E-01	8.97E-03	1.76E-01	7.78E-02	4.33E-01
Zn	5.25E-04	5.43E-04	6.37E-05	7.58E-04	3.37E-04	5.88E-04	6.08E-04	7.13E-05	8.49E-04	3.77E-04	1.11E-03
TL											
As	3.17E-02	2.70E-02	1.88E-02	1.51E-01	8.65E-03	5.33E-02	4.54E-02	3.16E-02	2.54E-01	1.45E-02	8.50E-02
Cd	5.34E-04	5.31E-04	1.29E-04	1.20E-03	1.80E-04	1.67E-01	1.66E-01	4.04E-02	3.75E-01	5.65E-02	1.68E-01
Cr	7.94E-05	7.77E-05	1.38E-05	1.27E-04	4.41E-05	1.37E-02	1.34E-02	2.37E-03	2.19E-02	7.60E-03	1.38E-02
Cu	1.06E-03	1.03E-03	3.12E-04	3.44E-03	2.81E-04	5.95E-03	5.76E-03	1.75E-03	1.93E-02	1.57E-03	7.01E-03
Hg	9.92E-04	7.54E-04	1.32E-03	1.85E-02	1.65E-04	2.78E-03	2.11E-03	3.69E-03	5.17E-02	4.63E-04	3.77E-03
Ni	2.71E-03	2.60E-03	7.42E-04	6.47E-03	1.00E-03	1.33E+00	1.27E+00	3.64E-01	3.17E+00	4.90E-01	1.33E+00
Pb	3.37E-01	3.35E-01	3.76E-02	5.40E-01	2.14E-01	1.13E-01	1.13E-01	1.26E-02	1.81E-01	7.18E-02	4.50E-01
Zn	4.68E-04	4.68E-04	9.95E-05	1.25E-03	2.01E-04	5.24E-04	5.25E-04	1.11E-04	1.40E-03	2.25E-04	9.92E-04
DZ											
As	3.67E-02	3.77E-02	1.62E-02	1.81E-01	9.76E-03	6.17E-02	6.33E-02	2.71E-02	3.03E-01	1.64E-02	9.84E-02
Cd	6.20E-04	5.70E-04	2.39E-04	2.60E-03	2.18E-04	1.94E-01	1.79E-01	7.50E-02	8.15E-01	6.84E-02	1.95E-01

Cr	8.43E-05	8.58E-05	1.03E-05	1.10E-04	4.75E-05	1.45E-02	1.48E-02	1.77E-03	1.89E-02	8.19E-03	1.46E-02
Cu	1.10E-03	1.10E-03	1.96E-04	1.67E-03	3.80E-04	6.15E-03	6.18E-03	1.10E-03	9.37E-03	2.13E-03	7.25E-03
Hg	7.75E-04	5.98E-04	5.86E-04	5.59E-03	1.37E-04	2.17E-03	1.68E-03	1.64E-03	1.56E-02	3.85E-04	2.95E-03
Ni	2.96E-03	2.98E-03	5.32E-04	4.20E-03	1.26E-03	1.45E+00	1.46E+00	2.61E-01	2.06E+00	6.16E-01	1.45E+00
Pb	3.47E-01	3.47E-01	3.44E-02	7.00E-01	2.59E-01	1.17E-01	1.16E-01	1.16E-02	2.35E-01	8.69E-02	4.64E-01
Zn	5.03E-04	5.07E-04	7.23E-05	8.38E-04	2.19E-04	5.63E-04	5.68E-04	8.09E-05	9.39E-04	2.45E-04	1.07E-03

Table S5. Estimations of non-carcinogenic (Hazard Quotient, HQ) for different exposure pathways from heavy metals in soil.

	ADI_{ing}x10^{-6} mg/kg/d		$ADIi_{nh}$ x10^{-9} mg/kg/d		ADI_{der}x10^{-7} mg/kg/d		HQ_{oral}		HQ_{dermal}		HI	
	Adult	Child	Adult	Child	Adult	Child	Adult	Child	Adult	Child	Adult	Child
As	1.832	10.266	1.078	1.147	31.328	17.247	6.11E-03	3.42E-02	1.04E-02	5.75E-02	1.66E-02	9.17E-02
Cd	0.098	0.548	0.058	0.061	7.802	4.295	9.78E-05	5.48E-04	3.12E-02	1.72E-01	3.13E-02	1.72E-01
Cr	22.273	124.805	13.102	13.949	507.822	279.563	1.49E-05	8.32E-05	2.60E-03	1.43E-02	2.62E-03	1.44E-02
Cu	7.964	44.628	4.685	4.988	453.973	249.919	1.99E-04	1.12E-03	1.13E-03	6.25E-03	1.33E-03	7.36E-03
Hg	0.023	0.127	0.013	0.014	0.646	0.356	1.42E-04	7.94E-04	4.04E-04	2.22E-03	5.45E-04	3.02E-03
Ni	10.398	58.263	6.116	6.512	2074.355	1141.962	5.20E-04	2.91E-03	2.59E-01	1.43E+00	2.60E-01	1.43E+00
Pb	8.231	46.123	4.842	5.155	28.151	15.497	5.88E-02	3.29E-01	2.01E-02	1.11E-01	7.89E-02	4.40E-01
Zn	25.939	145.347	15.258	16.245	295.703	162.789	8.65E-05	4.85E-04	9.86E-05	5.43E-04	1.85E-04	1.03E-03
THI											3.91E-01	2.16E+00

Table S6. Analytical quality assurances for the data
(a) Limit of detection (LOD)

Element	LOD (mg/kg)	Detection range (mg/kg)
As	0.2	0.2-500
Cd	0.02	0.02-4.0
Cr	3	3-3500
Cu	0.8	0.8-2000
Hg	0.0004	0.0004-10
Ni	1	1-2000
Pb	1.5	1.5-2000
Zn	1	1-3000
Co	0.04	0.04-500
Mn	5	5-2500
Mo	0.25	0.25-100
Sr	1	1-1200
Ti	5	5-46000
Sb	0.04	0.04-300
Sc	0.6	0.6-500
Se	0.008	0.008-100
Sn	0.5	0.5-100
SiO_2	0.5	0.5-900
Al_2O_3	0.1	0.1-300
TFe_2O_3	0.1	0.1-300

(b) Accuracy and precise for measurements (ΔlgC represents accuracy that is calculated from the differences between lgmean and lgRM; RSD (relative standard deviation) represents the ratio of standard deviation to mean)

Element		GSS1	GSS2	GSS3	GSS4	GSS5	GSS6	GSS7	GSS8	GSS-9	GSS10	GSS11	GSS12
As	Mean	34.1	13.4	4.16	59.7	418.6	212.8	4.5	13	8	9.1	7.1	11.3
	RM	33.5	13.6	4.4	58	412	220	4.8	12.7	8.4	8.9	7.4	12.2
	ΔlgC	0.008	-0.01	-0.02	0.013	0.007	-0.02	-0.03	0.01	-0.02	0.01	-0.02	-0.04
	RSD%	2.9	4.24	3.4	3.38	2.13	4.12	4.8	3.22	4.36	3.13	4.66	3.54
Cd	Mean	4.05	0.072	0.054	0.318	0.427	0.119	0.074	0.12	0.098	0.1	0.132	0.152
	RM	4.3	0.071	0.059	0.35	0.45	0.13	0.08	0.13	0.1	0.105	0.125	0.15
	ΔlgC	-0.03	0.006	-0.04	-0.05	-0.03	-0.04	-0.04	-0.04	-0.01	-0.02	0.024	0.006
	RSD%	3.26	4.6	4.51	4.5	4.91	4.19	4.99	3.04	4.21	4.66	4.26	3.55
Cr	Mean	67.2	50.5	34.2	383.6	120	81.1	433.8	62.8	71	61	62	54
	RM	62	47	32	370	118	75	410	68	75	58	59	59
	ΔlgC	0.035	0.031	0.029	0.016	0.007	0.034	0.025	-0.04	-0.03	0.022	0.022	-0.04
	RSD%	4.07	4	4.01	0.99	0.91	3.1	0.53	4.89	3.12	2.15	1.95	2.54

Cu	Mean	22.3	17	12	43	132.9	416.5	89.5	23.7	24.5	18.8	22	30.5
	RM	21	16.3	11.4	40.5	144	390	97	24.3	25	19	21.4	29
	ΔlgC	0.026	0.018	0.022	0.026	-0.04	0.029	-0.04	-0.01	-0.01	-0.01	0.012	0.022
	RSD%	1.69	4.35	3.38	1.35	0.45	1.66	0.72	2.59	2.32	2.13	2.09	3.22
Hg	Mean	0.034	0.016	0.057	0.603	0.282	0.073	0.057	0.016	0.035	0.032	0.056	0.022
	RM	0.032	0.015	0.06	0.59	0.29	0.072	0.061	0.017	0.032	0.033	0.06	0.021
	ΔlgC	0.026	0.028	-0.03	0.009	-0.02	0.006	-0.03	-0.03	0.039	-0.02	-0.03	0.02
	RSD%	4.48	4.78	4.85	2.68	3.49	2.51	4.9	4.28	4.23	4.32	4.66	4.12
Ni	Mean	20.6	19.3	11.7	65.1	41.5	52.3	284	30.3	31.3	25.3	27.2	33.6
	RM	20.4	19.4	12	64	40	53	276	31.5	33	26	25.4	32
	ΔlgC	0.004	-0.01	-0.01	0.007	0.016	-0.01	0.012	-0.02	-0.03	-0.02	0.03	0.021
	RSD%	1.97	2.98	3.89	1.09	1.38	0.95	0.31	1.27	1.01	0.95	1.05	1.32
Pb	Mean	94.3	20.3	26.6	57.4	553.5	306.5	13.5	20.6	24.6	21.3	25.9	20
	RM	98	20	26	58	552	314	14	21	25	22	24.7	19
	ΔlgC	-0.02	0.006	0.01	-0.01	0.001	-0.01	-0.02	-0.01	-0.01	-0.02	0.021	0.022
	RSD%	1.35	4.57	2.3	2.48	0.28	0.51	4.23	3.83	3.62	2.36	3.16	4.05
Zn	Mean	652.5	43.3	32.5	210.4	477.7	98	147.1	67.5	64.2	59.3	64.2	75.3
	RM	680	42	31	210	494	97	142	68	61	60	65	78
	ΔlgC	-0.02	0.013	0.021	0.001	-0.02	0.004	0.015	-0.01	0.022	-0.01	-0.01	-0.02
	RSD%	0.55	1.4	1.85	0.35	1.09	3.05	0.45	1.4	0.95	1.06	1.12	0.96
Co	Mean	14.5	8.74	5.7	21.6	11.9	7.3	101	13	14.5	11.3	11.55	13
	RM	14.2	8.7	5.5	22.3	12.3	7.6	97	12.7	14	11.7	11.6	12.6
	ΔlgC	0.009	0.002	0.016	-0.02	-0.02	-0.02	0.018	0.01	0.015	-0.02	-0.01	0.014
	RSD%	4.1	1.7	2.5	2.2	1.4	2.5	3.2	2.2	3.5	2.7	2.5	2.9
Mn	Mean	1720	514	304	1509	1356	1504	1708	639	501	694	589	791
	RM	1760	510	304	1420	1360	1450	1780	650	520	681	572	774
	ΔlgC	-0.01	0.003	0	0.026	-0.01	0.016	-0.02	-0.01	-0.02	0.008	0.013	0.009
	RSD%	0.65	0.49	1.17	0.56	0.43	0.39	1.36	1.23	2.1	1.33	3.25	2.36
Mo	Mean	1.46	0.96	0.29	2.46	4.76	17.04	2.78	1.23	0.42	0.49	0.57	1
	RM	1.4	0.98	0.3	2.6	4.6	18	2.9	1.16	0.4	0.52	0.6	0.96
	ΔlgC	0.018	-0.01	-0.02	-0.03	0.015	-0.03	-0.02	0.025	0.021	-0.03	-0.03	0.018
	RSD%	4.14	4.13	4.6	3.92	4.23	4.2	3.79	4.6	4.85	4.14	4.33	3.69
Sr	Mean	155.7	190.7	376	79.2	42.8	39.8	27.8	228.8	171	219	180	243
	RM	155	187	380	77	42	39	26	236	172	226	182	240
	ΔlgC	0.002	0.009	-0.01	0.012	0.008	0.009	0.029	-0.02	-0.01	-0.02	-0.01	0.005
	RSD%	0.36	0.46	0.28	0.75	1.18	1.74	3.19	0.47	2.32	2.98	3.65	2.54
Ti	Mean	5039	2955	2324	11200	6482	4638	20696	3884	4146	4156	4021	4033
	RM	4830	2710	2240	10800	6290	4390	20200	3800	4240	4270	3920	3920
	ΔlgC	0.018	0.038	0.016	0.016	0.013	0.024	0.011	0.009	-0.01	-0.02	0.011	0.012
	RSD%	0.39	0.39	1.24	0.43	0.43	0.83	0.39	0.9	1.03	0.98	1.11	1.32
Sb	Mean	0.83	1.33	0.44	6.07	36.61	55.93	0.44	1.03	0.86	0.71	0.62	1
	RM	0.87	1.3	0.45	6.3	35	60	0.42	1	0.85	0.68	0.61	1.05

	ΔlgC	-0.02	0.01	-0.01	-0.02	0.02	-0.04	0.02	0.013	0.005	0.019	0.007	-0.03
	RSD%	4.57	3.1	4.02	4.06	4.51	4.19	4.71	4.56	4.52	3.66	2.98	3.65
Sc	Mean	10.7	11	4.8	19.8	16.2	15.3	26.3	10.8	11.8	10.6	10.5	11.9
	RM	11.2	10.7	5	20	17	15.5	28	11.7	12.1	10.2	10	12.6
	ΔlgC	-0.02	0.012	-0.02	-0.01	-0.03	-0.01	-0.03	-0.04	-0.02	0.017	0.021	-0.03
	RSD%	4.23	4.88	4.85	2.18	5	2.74	4.91	3.35	3.56	4.31	4.98	4.73
Se	Mean	0.138	0.168	0.09	0.668	1.622	1.368	0.34	0.102	0.145	0.212	0.201	0.156
	RM	0.14	0.16	0.094	0.64	1.6	1.34	0.32	0.1	0.15	0.21	0.2	0.16
	ΔlgC	-0.01	0.021	-0.02	0.019	0.006	0.009	0.026	0.009	-0.02	0.004	0.002	-0.02
	RSD%	4.46	4.63	4.53	4.81	2.46	4.43	4.95	4.55	3.65	4.98	4.62	4.2
Sn	Mean	6.5	2.9	2.5	5.7	17.5	74.5	3.6	2.7	3.2	3.2	3.1	2.9
	RM	6.1	3	2.5	5.7	18	72	3.6	2.8	3.4	3.4	3.1	2.8
	ΔlgC	0.028	-0.02	0	0	-0.02	0.015	0	-0.02	-0.03	-0.03	0	0.015
	RSD%	4.72	4.73	3.83	3.96	4.39	4.66	3.81	4.83	3.25	4.63	3.29	4.66
SiO_2	Mean	64.32	71.09	73.92	49.69	52.3	54.92	34.04	56.61	60.65	64.88	70.09	60.98
	RM	62.6	73.35	74.72	50.95	52.57	56.93	32.69	58.61	61.69	65.5	69.42	60.01
	ΔlgC	0.012	-0.02	-0.01	-0.02	-0.01	-0.02	0.018	-0.02	-0.01	-0.01	0.004	0.007
	RSD%	0.22	0.11	0.34	0.46	0.51	0.21	0.32	0.83	0.36	0.65	0.45	0.63
Al_2O_3	Mean	14.06	10.73	11.82	23.34	21.53	22.15	28.01	11.69	13.58	13.65	13.25	13.24
	RM	14.18	10.31	12.24	23.45	21.58	21.23	29.26	11.92	13.28	13.8	13.14	13.27
	ΔlgC	-0.01	0.017	-0.02	-0.01	-0.01	0.018	-0.02	-0.01	0.01	-0.01	0.004	-0.01
	RSD%	0.86	0.48	1.77	0.38	0.69	0.15	0.3	0.91	0.62	0.85	0.35	0.51
Fe_2O_3	Mean	5.04	3.45	1.99	10.54	13.02	8.28	18.59	4.3	4.69	4.22	4.23	4.52
	RM	5.19	3.52	2	10.3	12.62	8.09	18.76	4.48	4.8	4.17	4.21	4.71
	ΔlgC	-0.02	-0.01	-0.01	0.01	0.014	0.01	-0.01	-0.02	-0.01	0.005	0.002	-0.02
	RSD%	0.57	0.57	0.4	0.42	0.43	0.41	0.18	0.82	0.62	0.36	0.62	0.36

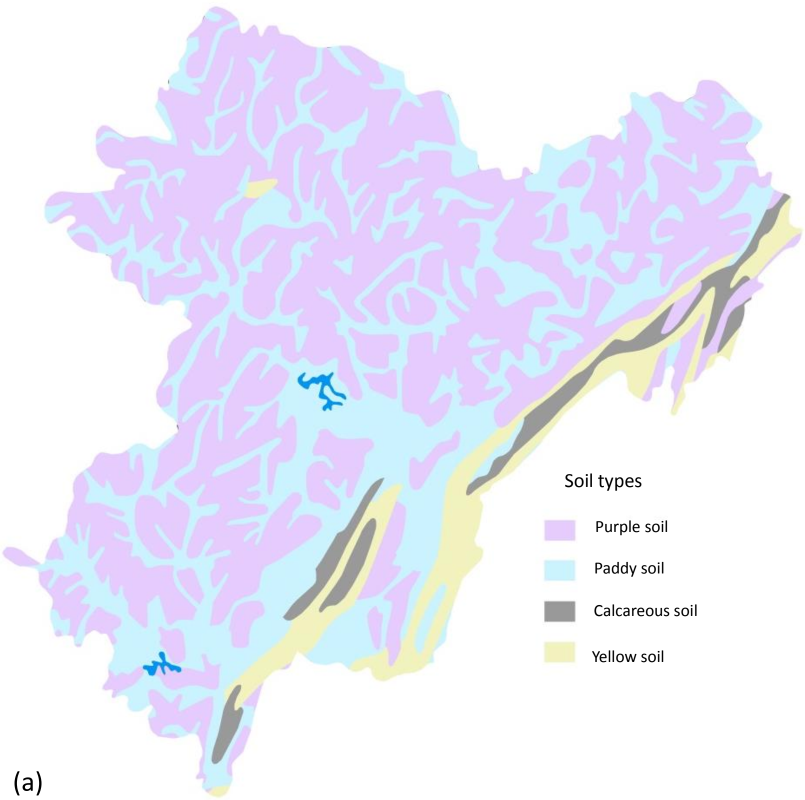

(a)

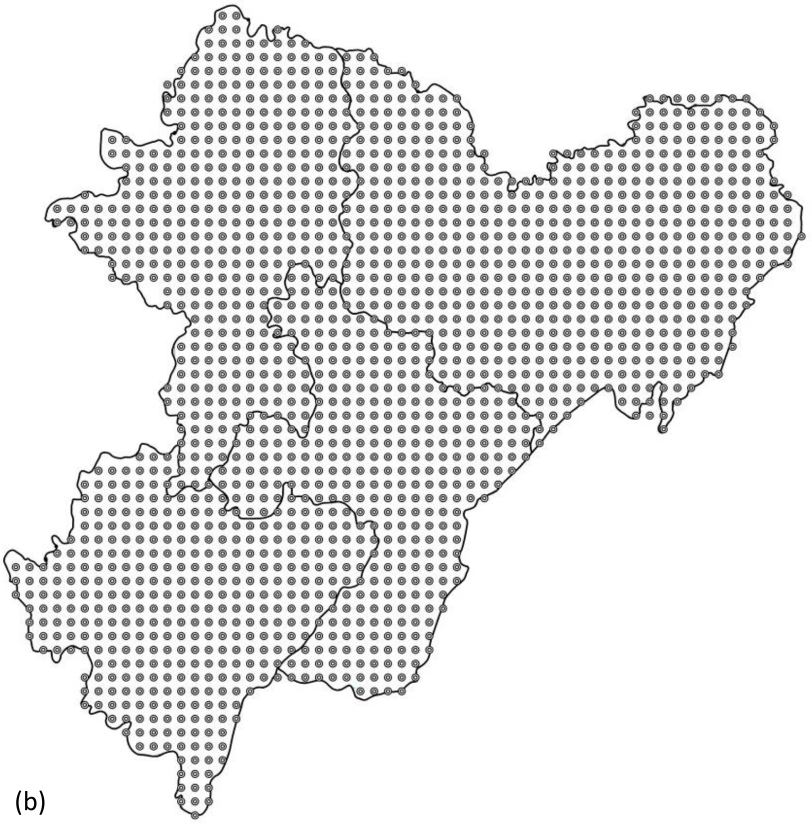

(b)

Fig. S1. Soil types (a) and sampling location (b) in the study area (a is provided by China Geological Survey via http://geocloud.cgs.gov.cn/appcloud/viewer/resourceList.html; b is from Data Center for Resources and Environmental Sciences, Chinese Academy of Sciences (RESDC),

http://www.resdc.cn; The ArcMap 10.3 software is used to create Fig. S1 with Figure legends and the software can be accessible *via* the link https://blogs.esri.com/esri/arcgis/2014/12/10/arcgis-10-3-the-next-generation-of-gis-is-here/).

References

Finley, B., Proctor, D., Scott, P., Harrington, N., Paustenbach, D., Price, P., 1994. Recommended distributions for exposure factors frequently used in health risk assessment. Risk Anal. 14, 533-553.

Jiang, Y., Chao, S., Liu, J., Yang, Y., Chen, Y., Zhang, A., Cao, H., 2017. Source apportionment and health risk assessment of heavy metals in soil for a township in Jiangsu Province, China. Chemosphere 168, 1658-1668.

Li, Z., Ma, Z., van der Kuijp, T.J., Yuan, Z., Huang, L., 2014. A review of soil heavy metal pollution from mines in China: Pollution and health risk assessment. Science of the Total Environment 468, 843-853.

MEPC, Ministry of Environmental Protection of the People's Republic of China, 2013. Exposure Factors Handbook of Chinese Population. China Environmental Science Press, Beijing, China.

USDoE., 2011.The Risk Assessment Information System (RAIS). U.S. Department of Energy’s Oak Ridge Operations Office (ORO).

USEPA (US Environmental Protection Agency), 2001. Supplemental guidance for developing soil screening levels for superfund sites. OSWER9355.4-24. Office of Solid Waste and Emergency Response. US Environmental Protection Agency. Washington, DC.

USEPA, 2011. Exposure Factors Handbook 2011 Edition (Final). U.S. Environmental Protection Agency, Washington, DC, EPA/600/R-09/052F.

HC (Health Canada). Federal Contaminated Site Risk Assessment in Canada-Part II: Health Canada Toxicological Reference Values (TRVs) and Chemical-Specific Factors [R]. Ottawa, Canada; 2004.
